# The migratory pathways of the cells that form the endocardium, dorsal aortae, and head vasculature in the mouse embryo

**DOI:** 10.1186/s12861-021-00239-3

**Published:** 2021-03-22

**Authors:** C. Collart, A. Ciccarelli, K. Ivanovitch, I. Rosewell, S. Kumar, G. Kelly, A. Edwards, J. C. Smith

**Affiliations:** 1 Developmental Biology Laboratory, Francis Crick Institute, 1 Midland Road, London, NW1 1AT UK; 2 Advanced Light Microscopy Facility, Francis Crick Institute, 1 Midland Road, London, NW1 1AT UK; 3 Genetic Modification Service, Francis Crick Institute, 1 Midland Road, London, NW1 1AT UK; 4 Photonics Group, 606 Blackett Laboratory, Imperial College London, South Kensington Campus, London, SW7 2AZ UK; 5 Bioinformatics and Biostatistics Facility, Francis Crick Institute, 1 Midland Road, London, NW1 1AT UK; 6 Advanced Sequencing Facility, Francis Crick Institute, 1 Midland Road, London, NW1 1AT UK

**Keywords:** SCL/Tal1, Circulatory system, VEGF, Apela, Endothelial cell, Mesoderm, Vasculogenesis, Endocardium, Dorsal aorta, Head vasculature

## Abstract

**Background:**

Vasculogenesis in amniotes is often viewed as two spatially and temporally distinct processes, occurring in the yolk sac and in the embryo. However, the spatial origins of the cells that form the primary intra-embryonic vasculature remain uncertain. In particular, do they obtain their haemato-endothelial cell fate in situ, or do they migrate from elsewhere? Recently developed imaging techniques, together with new Tal1 and existing Flk1 reporter mouse lines, have allowed us to investigate this question directly, by visualising cell trajectories live and in three dimensions.

**Results:**

We describe the pathways that cells follow to form the primary embryonic circulatory system in the mouse embryo. In particular, we show that Tal1-positive cells migrate from within the yolk sac, at its distal border, to contribute to the endocardium, dorsal aortae and head vasculature. Other Tal1 positive cells, similarly activated within the yolk sac, contribute to the yolk sac vasculature. Using single-cell transcriptomics and our imaging, we identify VEGF and Apela as potential chemo-attractants that may regulate the migration into the embryo. The dorsal aortae and head vasculature are known sites of secondary haematopoiesis; given the common origins that we observe, we investigate whether this is also the case for the endocardium. We discover cells budding from the wall of the endocardium with high Tal1 expression and diminished Flk1 expression, indicative of an endothelial to haematopoietic transition.

**Conclusions:**

In contrast to the view that the yolk sac and embryonic circulatory systems form by two separate processes, our results indicate that Tal1-positive cells from the yolk sac contribute to both vascular systems. It may be that initial Tal1 activation in these cells is through a common mechanism.

**Supplementary Information:**

The online version contains supplementary material available at 10.1186/s12861-021-00239-3.

## Background

Blood vessel formation in amniotes is usually described as consisting of two spatially and temporally distinct processes: extra-embryonic vasculogenesis and intra-embryonic vasculogenesis [1–3]. This view derives from embryological experiments carried out a hundred years ago in bird embryos [4, 5], and has been generally accepted for amniotes. Vasculogenesis has been much studied between then and now, with techniques including static imaging, transplantation assays, lineage tracing, and single-cell transcriptomics, but in mammalian embryos cell movement has been difficult to observe directly, leaving a gap in our understanding. In particular, in the mouse, the spatial origins of the cells that form the intra-embryonic vasculature remains unknown, leaving it unclear how much commonality there is between extra-embryonic and intra-embryonic vasculogenesis. The migratory pathway of these cells is also important because it defines their tissue environment as they become specified.

Here we focus on how the endothelium of the primary circulatory system begins to form in the mouse embryo, including that of the endocardium, the dorsal aortae, and the head vasculature. Recently-developed imaging techniques allow us to investigate this process directly, visualising cell trajectories in detail and in three dimensions, with both live and still imaging. To label cells that give rise to the endothelium of the vasculature, we use two fluorescent reporter mouse lines: a newly developed Tal1/SCL-cerulean strain and an existing Flk1/Kdr-GFP strain [6]. We study the expression of Tal1 and Flk1 in detail between E7/E7.25 and E8 in the whole embryo, and also specifically in the endocardium at E9.5. Our imaging data inspired us to carry out a single-cell transcriptomics experiment, and we use these data, together with the imaging, to identify candidate chemo-attractants that may be involved in patterning of the primary circulatory system.

Tal1, a basic helix-loop-helix transcription factor, is a frequently-used marker for haemogenic endothelium [7, 8], and is expressed at early stages during specification of the mesoderm [9]. Tal1 knock-out mice die, having failed to develop blood, at E9.5 [10]. The database and lineage tree obtained by single cell transcriptomics in [8] show that between E7.25 and E8, Tal1 first labels haemato-endothelial progenitors and blood progenitors and then, in addition, erythrocytes and endothelium. Blood progenitors, erythrocytes, and endothelium were found to be derived from haemato-endothelial progenitors. CD41-positive blood progenitor populations are localised in the proximal end of the yolk sac [11]. During this period, Tal1 is a key regulator in mesoderm specification. It suppresses alternative lineages in blood-fated cells [12]; it has been implicated in lineage restriction of cardiovascular progenitors; and it represses expression of cardiac-specific regulators and structural proteins to prevent ectopic cardiac activity [13]. Tal1 is essential for specification of the three haematopoietic waves, the maturation of select blood lineages, and the remodelling of the vascular network during development [13]. Our results shed light on the early expression pattern of Tal1 in great detail.

The second marker, Flk1 (also known as vascular endothelial growth factor receptor-2, VEGFR-2) is expressed in vascular and hematopoietic progenitors and is thought to function upstream of Tal1 [14]. However, at early stages Flk1 is not specific for angioblasts or endothelial cells—it also marks cells that give rise to myocardium and skeletal muscle—and it is not clear when its expression becomes restricted to them [6, 15]. This is why we developed the new Tal1 reporter strain.

## Results

### Formation of the dorsal aortae, endocardium and head vasculature involves migration of Tal1 positive cells from the distal border of the yolk sac

To visualise the distribution and movement of the cells that give rise to the endothelium of the vasculature, we first studied Tal1 expression at the cellular level in three dimensions. To visualise Tal1, we created a Tal1-cerulean mouse line (materials and methods). Tal1 and cerulean are separated by a self-cleaving peptide, so as to not affect the function of Tal1, and cerulean is tagged with a nuclear localisation signal. Uncleared fixed embryos between E7.25 and E8 were imaged at closely-spaced time points using light sheet microscopy, and cleared embryos at E9.5 were imaged by optical projection tomography. All embryos are shown rotating in three dimensions as supplemental movies (Additional Files 2,3, 4, 5, 6, 7, 8, 9, 10, 11 and 12), and brightfield images are shown in Additional File 1. To understand how cells migrate during development we also imaged live embryos from approximately E7.5, using both light sheet and two-photon microscopy (also in Additional File movies). These movies are essential to interpret our data; we have included key snapshots in the still figures, but these cannot completely convey the 3D and temporal structures involved. To make it easier to access the movies, we include links. In what follows we first describe data obtained using fixed embryos, and then go on to describe the results of live imaging.

#### Imaging fixed embryos using light sheet microscopy and optical projection tomography

To establish the detailed expression pattern of Tal1 in the initial vasculature, we show still imaging results.

**Fig. 1:**
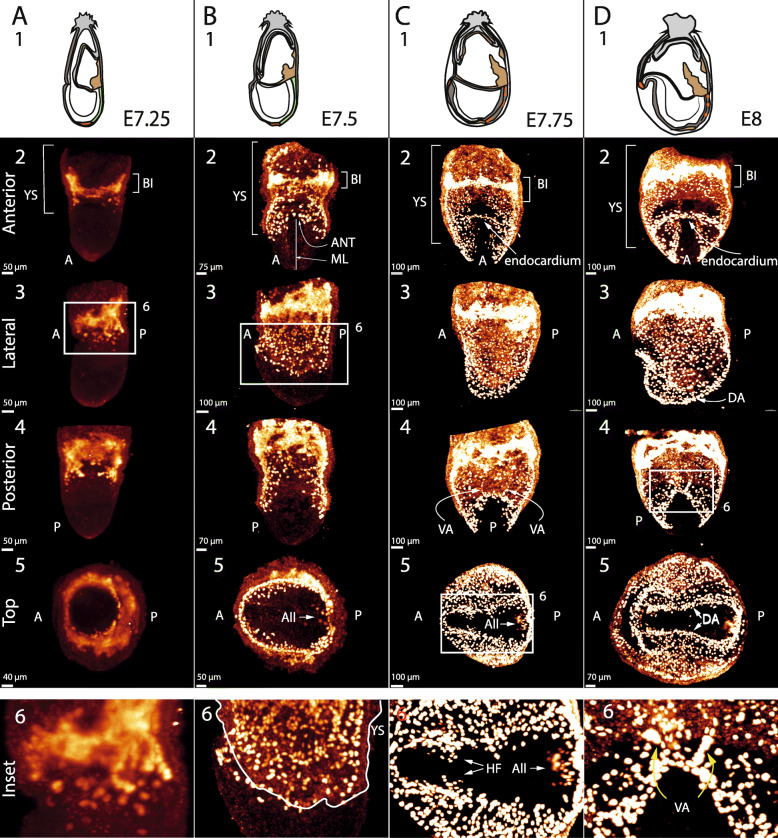
Formation of the endocardium, dorsal aortae, and head vasculature. A = anterior, P = posterior, BI = blood islands, YS = yolk sac, ML = midline, ANT = anterior neural tube, All = allantois, HF = head folds, DA = dorsal aortae, VA = vitelline arteries. The subfigures show Tal1 expression in fixed uncleared embryos, imaged by light sheet microscopy. (**a1–6**), (**b1–6**), (**c1–6**), (**d1–6**): schematic representation (as in the Kaufman’s atlas of mouse development), anterior view, side view, posterior view, top view, and enlarged inset of E7.25, E7.5, E7.75, and E8 embryos. The horizontal series (**a2, b2, c2, d2**), (**a3, b3, c3, d3**), (**a4, b4, c4, d4**), (**a5, b5, c5, d5**) show, respectively, the onset of the formation of the endocardium, dorsal aortae, vitelline arteries, and head vasculature over time. (**a6**): enlarged inset of A3 depicting Tal1+ cells in a speckled pattern underneath the BI. (**b6**): enlarged inset of B3, showing prospective endothelial cells outside the YS at the anterior part of the embryo. (**c6**): enlarged inset of C5 showing Tal1+ cells that ingressed into the head folds and allantois. (**d6**): enlarged inset of D4 showing the vitelline arteries developing

At E7.25 (https://youtu.be/6j0kVo68Trw, Additional File 2, Additional File 1A and Fig. 1A1-6) Tal1 is expressed in the developing blood islands (Fig. 1A2), with some Tal1-expressing cells also seen beneath the blood islands in a speckled pattern in the yolk sac (Fig. 1A3,6).

At E7.5 (https://youtu.be/KlTt4AuXLxs, Additional File 3, S. https://youtu.be/BlJSeiY8H9E, Additional File 4, Additional File 1B and Fig. 1B1-6) Tal1 positive cells occupy the entire yolk sac and the bud of the allantois, again in a speckled pattern but with many more cells (Fig. 1B5). Some cells can now also be observed just outside the yolk sac, in the embryonic region near the anterior part of the developing neural tube (Fig. 1B3,6). At this stage the Tal1 positive cells are blood progenitors or haemato-endothelial progenitors [8]. Ferkowicz et al. [11] show that the former (there identified as CD41-positive) are localised in the proximal end of the yolk sac, which corresponds with the very bright zone of Tal1 positive cells in the yolk sac that we label as the blood islands (BI) (Fig. 1B2). We later see that Tal1+ cells outside the yolk sac become part of the vasculature and so for convenience we refer to the Tal1 positive cells outside the yolk sac in our movies as prospective endothelial cells (they retain haematopoietic potential).

To observe the interior of the embryo more clearly, we dissected and imaged the anterior part of an embryo at E7.5 (https://youtu.be/YgWjGncTCQY, Additional File 5). This shows the Tal1 positive cells absent from a zone either side of the midline.

By E7.75 Tal1 positive cells are seen throughout the embryo, in addition to the yolk sac, except for a zone either side of the midline (https://youtu.be/v4YYS1npR9s, Additional File 6, https://youtu.be/XnUzi99nx7E, Additional File 7, Additional File 1C and Fig. 1C1-6).

At E7.75 we also observe Tal1 positive cells that have ingressed into the nascent head folds (Additional File 6 and Additional File 7 as above, and Fig. 1C5,6), marking the onset of the formation of the head vasculature. The anterior intestinal portal (AIP) has now formed underneath Tal1 positive cells that were previously in the vicinity of the anterior part of the neural tube (ANT in Fig. 1B2). The AIP is a pocket-like structure that is formed by the invagination of the outer layer of the embryo, the endoderm, which also pulls the anterior part of the neural tube inwards. The Tal1 positive cells above the AIP start forming a crescent that will become the endocardium (Additional File 6 and Additional File 7 as above, and Fig. 1C2).

At E8 (https://youtu.be/WSsIkmVeOLI, Additional File 8, https://youtu.be/tMD8hU6bfVU, Additional File 9; Additional File 1D, and Fig. 1D1-6), cords of prospective endothelial cells begin to form the dorsal aortae on either side of the midline along the entire border of the Tal1 cell free zone (Fig. 1D3,5). As in all amniote embryos, the definitive dorsal aorta forms as bilateral vessels that later fuse at the midline. Meanwhile, more cells are now contributing to the endocardium, which is becoming a pronounced crescent (Fig. 1D2), and more Tal1 positive cells are also visible in the head folds and the allantois (Additional File 9 again). At the same time, the omphalomesenteric system (the vasculature connecting the yolk sac with the embryo) starts to develop: the vitelline arteries at the distal end of the yolk sac, which will later connect the back of the dorsal aortae with the blood islands, begin to form (Fig. 1D4,6).

**Fig. 2:**
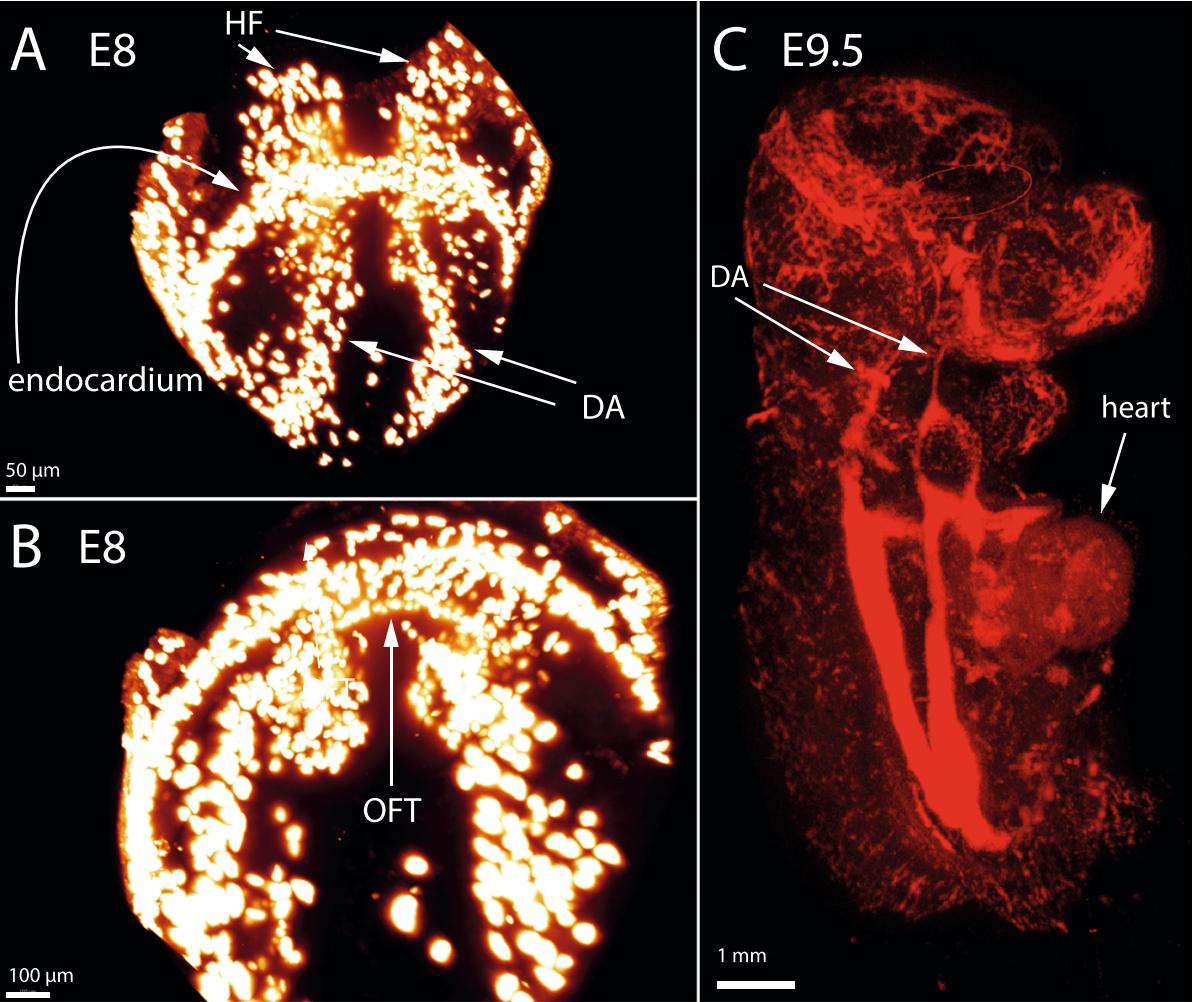
Formation of the head vasculature. The subfigures show Tal1 expression in fixed uncleared embryos, imaged by light sheet microscopy (**a,b**) and OPT (**c**). **a**: anterior view of the dissected anterior part of an E8 embryo showing that the dorsal aortae extend into the headfolds underneath the endocardium. **b**: bottom view of the anterior part of an E8 embryo, showing that the outflow tract of the endocardium starts to form as a semi-circle around the head folds. **c**: Side view of an E9.5 embryo, showing the dorsal aortae extending into the headfolds, past the heart

To observe the Tal1 positive cells in the head folds, which are hidden behind the heart tube and the yolk sac, we imaged the dissected anterior part of two E8 embryos (https://youtu.be/r_e-8owPxJ4, Additional File 10 and https://youtu.be/2-AcYc35120, Additional File 11, Additional File 1E, Fig. 2a). These images show that the dorsal aortae extend into the head folds. Cells at the back of the endocardium form a semicircle around the head folds (Fig. 2b). This will later become the outflow tract of the endocardium. The endocardium and the dorsal aortae are not yet connected (Fig. 2b).

Finally, at E9.5, optical projection tomography on cleared embryos reveals that the dorsal aortae still extend into the head. The endocardium is connected to the dorsal aortae via arches (https://youtu.be/h6a4mgedyLI, Additional File 12, Additional File 1F, Fig. 2c).

#### Imaging live embryos using light sheet and two-photon microscopy

A priori, the activation of Tal1 induction in the cells that form the embryonic vasculature could be either (a) entirely in situ, distinct from the Tal1 induction within the yolk sac, or (b) take place partly or entirely within the yolk sac, with Tal1 positive cells migrating from there to form the embryonic vasculature. To resolve this, we directly observe cell movement at a much finer temporal resolution, with live 3D imaging every 11 min of Tal1 and Flk1-labelled embryos, described here and in the next section respectively. This enables tracking of individual cell motion.

First, making use of a new non-invasive mounting protocol (Additional Files 13, 14 and 15), we imaged a live Tal1-labelled embryo at E7.5 for 9 h 43′ (https://youtu.be/NHEtAz1LSc8 = Additional File 16) by light sheet microscopy, a period covering the initial formation of the dorsal aortae and endocardium.

Some of the prospective endothelial cells that form the dorsal aortae can be seen within this time period to migrate from within the distal edge of the yolk sac. To document this, we manually tracked a number of these Tal1 positive cells, using the 3D data at each frame to resolve any ambiguities (https://youtu.be/gWRxDB-usj8 = Additional File 17, Fig. 3A1-3). That the cells are actively migrating, not passively following the morphogenetic movements of the developing embryo, is clear from the fact that the endothelial cells move with respect to each other, and the fact that the Tal1-free gap between the yolk sac and the forming dorsal aortae widens.

**Fig. 3:**
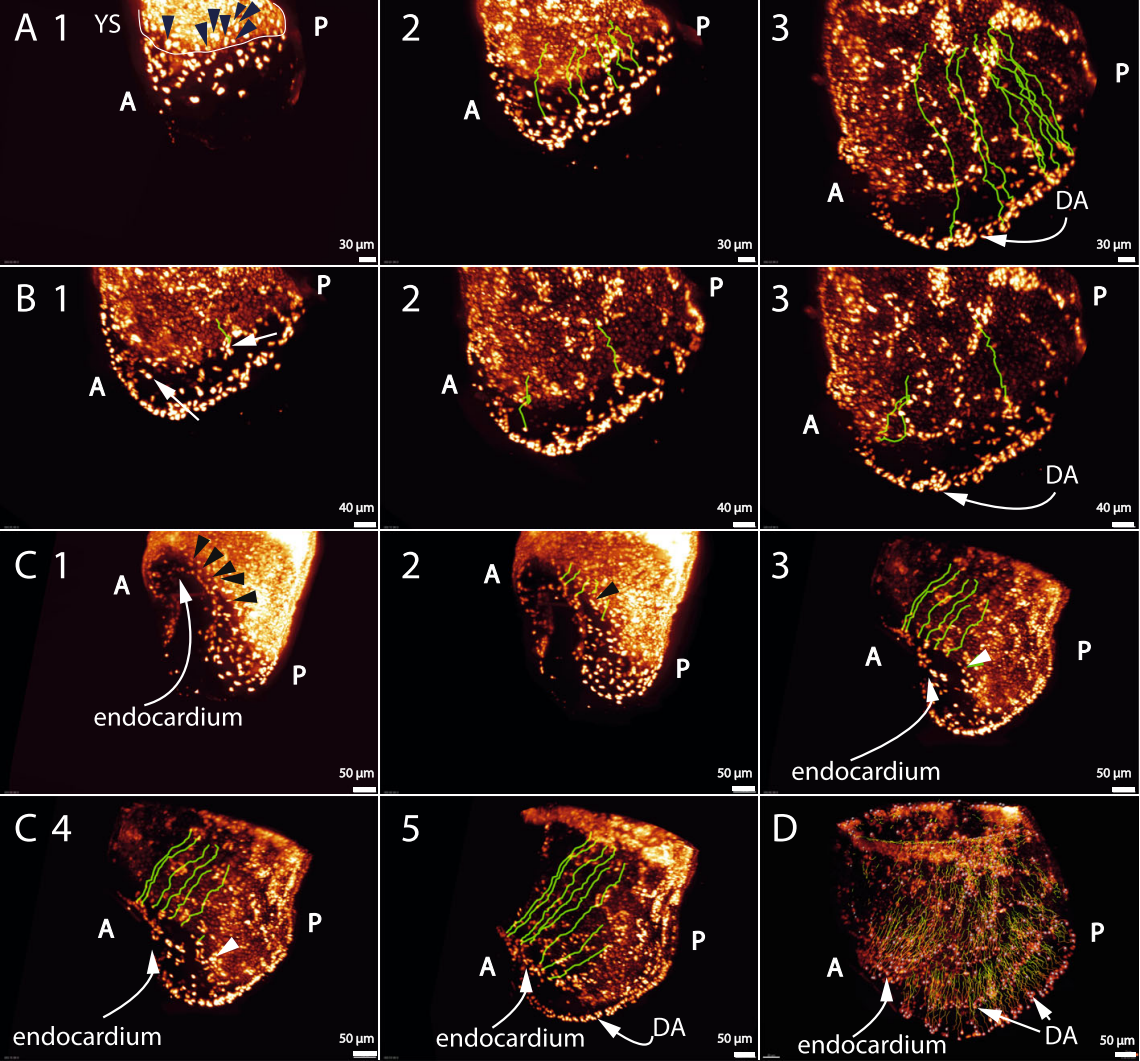
Prospective endothelial cells migrate from the yolk sac to form the dorsal aortae and endocardium. A = anterior, P = posterior, DA = dorsal aortae, YS = yolk sac. The subfigures all show Tal1 expression in frames of movies of live imaging time series, imaged by light sheet microscopy, together with the tracks of particular individual cells. (**a1–3**), (**b1–3**), (**c1–5**), and (**d**): stills from Additional File 17, Additional File 18, Additional File 19 and Additional File 20 respectively, showing that cells from within the distal border of the yolk sac migrate into the embryo to form the dorsal aortae (**a1–3**), that some endothelial cells return to or remain in the yolk sac (**b1–3**), and that cells at the border of the yolk sac migrate into the embryo to form the endocardium (**c1–5**). (**a1**): black arrows indicate single cells within the distal edge of the yolk sac that were tracked. (**a2,3**): green lines show the path along which the cells moved. Because the embryo also grows while the cells are migrating, the origin of the line no longer represents the origin of the cell migration inside the embryo shown, but the end of the line does indicate where the cells are at that timepoint. The lines represent the total displacement of the cells, which is the sum of morphogenetic movement and active migration. (**a3**): green tracking lines show that the migrating cells reached the forming dorsal aortae, on the distal side of the avascular zone forming between the yolk sac and the dorsal aortae. (**b1**): a white arrow on the left points to a cell within the embryo that was tracked, and a white arrow on the right points at a cell at the border of the yolk sac that was tracked (starting from an earlier time point) from within the yolk sac and moved along the green line. (**b2–3**): these show that the former cell divides and that its two daughter cells migrate to join the yolk sac, at different timepoints, and that the latter cell remained within the yolk sac, at its distal border. (**c1–5**): black and white arrows indicate cells within the distal border of the yolk sac, that migrate to contribute to the endocardium. The green lines represent the tracking lines as before. Cells that join the endocardium early come from the more anterior part of the yolk sac; cells that join later come from the lower down sideways edge of the yolk sac and join the venous poles. (**d**): this shows the direction of the cell movement of all the Tal1 positive cells in the embryo. This (**d**) tracking was automatic, while the (A-C) tracking was manual

We also identified and tracked a single prospective endothelial cell that divides into two daughter cells that both move back to join the yolk sac, and another that never leaves the yolk sac and instead clusters with other prospective endothelial cells at the edge of the yolk sac (https://youtu.be/FGd6IOGxljg = Additional File 18, Fig. 3B1-3). During this period Tal1 positive cells within the yolk sac are also clustering into cords.

Turning to the endocardium, we identified and tracked single cells that migrated from the yolk sac-embryo border to contribute to the endocardium (https://youtu.be/uG8DUSb74NA = Additional File 19, Fig. 3C1-5). As time progresses, cells that contribute to the crescent of the endocardium come from lower parts of the edge of the yolk sac, and form the venous poles of the endocardium.

For reference, we also show the overall movement of Tal1 positive cells, which is the result of the combination of active migration and the morphogenetic movements of the embryo (https://youtu.be/9eTl6dzuVvM = Additional File 20, Fig. 3d).

**Fig. 4:**
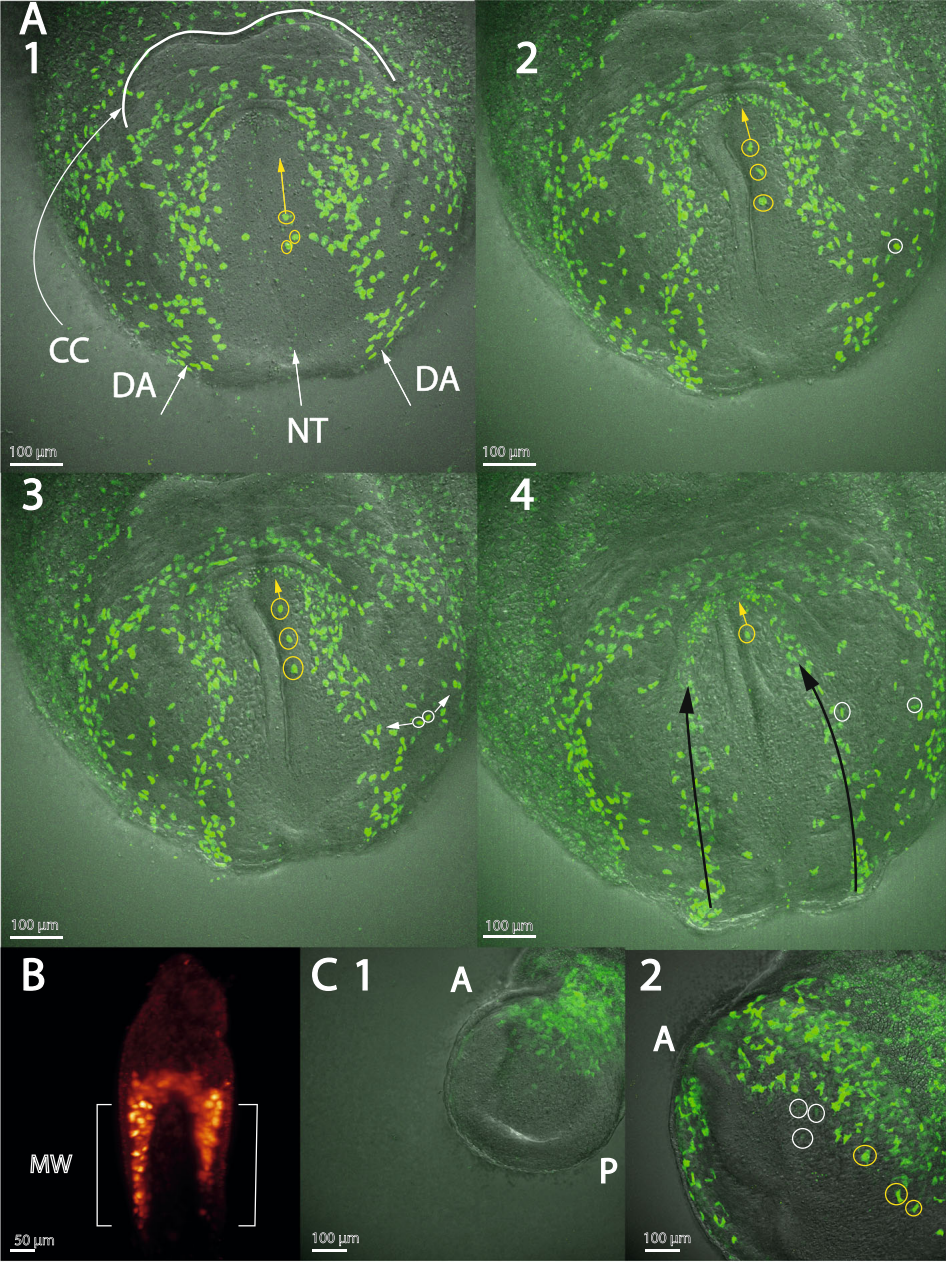
The dorsal aortae move underneath the endocardium; migrating endothelial cells enhance Flk1 expression and are plastic during migration. CC = cardiac crescent, DA = dorsal aortae, NT = neural tube, A = anterior, P = posterior, MW = mesodermal wing. (A1–4) and (C1–2): single frames of live imaging time series from Additional File 21 and Additional File 28, showing Tal1 and Flk1 expression respectively, imaged by two-photon microscopy. (**a1–4**): yellow circles indicate Tal1+ cells that migrate above the neural tube and yellow arrows show the direction of the cell movement. (**a2**): white circle indicates a Tal1+ cell that is about to divide. (**a3–4**): the daughter cells migrate in opposite directions (white arrows); one joins the dorsal aorta and the other the venous pole of the endocardium. (**a4**): black arrows indicate the direction of the cell flow in the dorsal aortae. (**b**): Flk1 expression in an E7 fixed, non-cleared embryo, imaged by light sheet microscopy, posterior view showing Flk1 expression in the yolk sac, as well as the mesodermal wings in the embryo. (**c1**): this shows an embryo, similar to the one in (**b**), with Flk1 expression in the embryo as well as in the yolk sac. (**c2**): this shows that migrating endothelial cells (recognisable by their shape, and by their motion against the background in the movie) increase their Flk1 expression (some of these have been circled in yellow), while other cells in the embryo (some circled in white) decreased their Flk1 expression

To improve our understanding of the formation of the head vasculature, where we had previously shown that the dorsal aortae extend into the headfolds, we next imaged the anterior region of an E7.75 embryo for 8 h by two-photon microscopy (https://youtu.be/XSt336cTv80 = Additional File 21). We see four main cell movements. Firstly, we observed a few cells migrating while above the neural tube (Fig. 4A1-4) (there is also some morphogenetic movement, but the differential speed of the cells with respect to the background indicates that they are migrating). These cells would be expected to be protected from any repulsive signals coming from the notochord beneath [16].

Secondly, the cells that are present between the dorsal aortae and the venous poles of the heart migrate from this area to join one or other of these structures. We observed two dividing cells in this region for which the daughter cells migrate in different directions, joining different structures, showing that the cells are plastic at this stage (Additional File 21 as before, Fig. 4A2-4). Thirdly, we see that the cells forming the dorsal aortae move into the anterior intestinal portal underneath the endocardium, by a combination of morphogenetic movements and cell migration, indicating that cells move directly from the dorsal aortae into the headfolds (Additional File 21 as before, Fig. 4A4). Finally, we observe that cells join the endocardium via the venous poles of the heart (Additional File 21 as before).

We conclude from this that the activation of Tal1 in cells that form the embryonic vasculature does take place partly or entirely within the yolk sac, with Tal1 positive cells migrating from within the distal border of the yolk sac to contribute to the endocardium, dorsal aortae, and head vasculature. Some Tal1 positive cells were already in the embryo at the start of the live-imaging video, so we cannot make definite conclusions about the origin of those, but we did not see any appearing de novo in the area between the yolk sac and the forming dorsal aorta where the cells are migrating, making it unlikely that they were activated there. Ideally one would resolve this question by live imaging from an earlier point, but the high fluorescent background generated in the yolk sac at early developmental stages in the embryo growth medium makes that challenging.

### Flk1 becomes specific for and is increasingly expressed in cells migrating to form the embryonic vasculature

To confirm our Tal1 results showing migration of prospective endothelial cells to form the embryonic vasculature, and to start investigating the mechanisms that may control this, we studied Flk1, whose ligand VEGF is already known to attract angioblasts to the midline in Xenopus [17], to be important for localisation of Flk1 positive cells in the anterior part of mouse embryos [18], and to control migration of mouse angioblasts in vitro [18]. The spatio-temporal expression pattern of VEGF shows that at E7.5, when migration of the Tal1 positive cells starts, VEGF is highly expressed in the yolk sac, but it also becomes visible in the anterior part of the embryonic endoderm, along the lines where the dorsal aortae start to form, (Fig. 2, f, g) in [19].

We used a Flk1-GFP reporter mouse, first imaging a series of non-cleared embryos by light sheet microscopy. At E7, Flk1 is expressed in the extra-embryonic mesoderm as well as in the embryonic mesodermal wings in cells with multiple identities (https://youtu.be/70BLmkcGybk = Additional File 22, Additional File 1G and Fig. 4b). At E7.5, E7.75, and E8, Flk1 expression resembles that of Tal1 in the dorsal aortae, yolk sac, and endocardium (https://youtu.be/o8vf66IxEcw, https://youtu.be/68GIuprmp6A, https://youtu.be/A5hcGvIYrAU, Additional File 23, 24, 25; Additional File 1H, I, J). The blood islands are not visible in the Flk1 expression pattern, while the Tal1 expression, occurring also in blood progenitors and the primitive blood, highlights them strongly. At E8, endothelial cells in the yolk sac start forming the vitelline veins. These are part of the omphalomesenteric system, connecting the endocardium with the yolk sac (Additional File 25 again). We see by light sheet microscopy on cleared samples at E9 the two separate blood vessels of the unfused dorsal aortae (https://youtu.be/VmRjgPxiGN4, Additional File 26; Additional File 1 K), and at E9.5 the start of fusion at the midline, from the middle of the embryo to the tail (https://youtu.be/rAmk5SWUGDc, Additional File 27; Additional File 1L).

We then live-imaged an E7 Flk1-expressing embryo by two-photon microscopy (https://youtu.be/cHc5LwYiHtg, Additional File 28 and Fig. 4C1-2). This shows that Flk1-expressing prospective endothelial cells start migrating towards the midline, confirming our Tal1 observations. This migration is recognisable by cell motion with respect to the background texture and by cell shape changes. Interestingly, the migrating cells increase their Flk1 expression, while expression in the others declines. This identifies the point at which Flk1 expression becomes specific for endothelial cells.

Our data add to the evidence that the Flk1 ligand VEGF contributes to the control of the migration in mouse. The knock-out of VEGF-A is haplo-insufficient in mouse embryos, making it difficult to study that question more directly [20].

### Single-cell transcriptomics identifies Apela as a potential chemo-attractant for cells migrating to form the embryonic vasculature

**Fig. 5:**
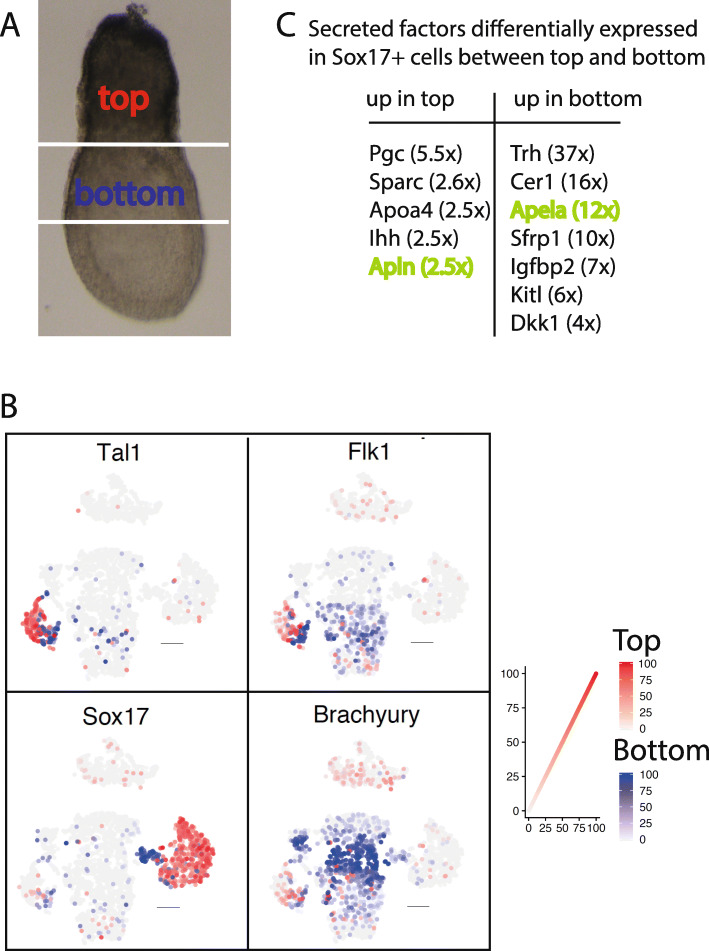
Differential Apela and Apelin expression in the endoderm. (**a**): the ‘top’ and ‘bottom’ parts of dissected embryos used for single-cell transcriptomics. (**b**): Expression of Tal1, Sox17, Flk1 and Brachyury (T) mapped onto t-distributed stochastic neighbour embedding (t-SNE) plots, showing the aggregate of all cells of ‘top’ and ‘bottom’ parts (as shown in **a**) of twenty-five (E7.25-E7.5) embryos in grey, the localisation of gene expression in the cells of the ‘top’ part in red and of the ‘bottom’ part in blue. The intensity of the colour represents the expression levels of the genes in these cells. (**c**): Secreted factors that are at least two-fold differentially expressed between Sox17 expressing cells of the ‘top’ sample versus the ‘bottom’ sample. The difference in fold expression is indicated between brackets. In green are indicated the secreted factors for which the receptor is expressed in Tal1+ cells

To identify additional potential chemo-attractants for migrating prospective endothelial cells, we dissected 25 embryos between E7.25 and E7.5 into the proximal bulk of the extra-embryonic region (designated ‘top’ for brevity), where Tal1 is expressed, and the extra-embryonic/embryonic border region immediately distal from that (designated ‘bottom’), into which Tal1 positive cells are migrating (Fig. 5a). We excluded the most distal part of the embryo so that our subsequent single-cell transcriptomic analysis (Additional File 29; Additional Files 30, 31) would be confined to these immediately adjacent regions. To validate gene expression in our experiment, we mapped the expression of Tal1, Flk1, Sox17 and Brachyury (T) onto the aggregate of cell clusters of top and bottom samples (Fig. 5b). Consistent with [8], we see Tal1 expressed in a subset of the clusters in which Flk1 is expressed, Sox17 expressed largely in a different set of clusters, and Flk1 expressed in a subset of the clusters in which Brachyury is expressed.

The endoderm is a well-known signalling centre for endothelial cells [21] and for vasculogenesis [3]. At these early stages Sox17 is mainly expressed in endoderm [8]. We therefore listed the genes that were differentially expressed between the ‘top’ and ‘bottom’ regions of the embryo, in cells that expressed Sox17 (Additional File 32). From this list, we went on to select secreted factors that showed at least a two-fold difference in expression levels between the two regions (twelve genes, Fig. 5c). We then asked whether the receptors for these ligands are expressed in Tal1+ cells. Of the receptors for the twelve genes, only the receptor Aplnr proved to be strongly expressed in Tal1+ cells, 6.8x and 3.4x upregulated in the two Tal1 expressing clusters compared to the mean Aplnr expression in all cells (clusters 5 and 8 in Additional file 29, 30 and 31). This binds Apelin and Apela, which are differentially expressed 2.5x up in `top’ and 12x up in `bottom’ respectively. Our data also identified other factors (Ihh, Sparc, Kitl) that are differentially expressed in the endoderm of top and bottom sample and that are known to be involved in haematopoiesis, but the Tal1+ cells did not express receptors for these factors at this timepoint.

Apela (also named Elabola, Ende, Toddler) is a strong candidate for a potential chemo-attractant in the mouse embryo. Its expressed in the definitive endoderm beneath the cardiac crescent, in the midline of the embryo, and in the base of the allantois [22]—all sites to which endothelial cells are attracted. Knock-out of Apela in the mouse embryo causes cardiovascular defects, albeit with low penetrance [23]. In addition, Helker et al. [24] show that in zebrafish Apela is needed to guide angioblasts to the right part of the embryo during embryogenesis, although in embryos where there is not enough Apela, Apelin can compensate for this deficiency and the first blood vessels will later develop correctly. Apelin has not only been shown to promote haematopoiesis from human embryonic stem cells [25], but also to promote chemotaxis in vitro for primary human endothelial cells [26]. The expression of Apelin is stronger in the top part and Apela expression is stronger in the bottom part at the timepoint we took our samples. However, at E8.5, Apelin (Supplementary figure in [26]) is also highly expressed in the areas in the mouse embryo where the endothelial cells have been attracted to.

The above literature implicates VEGF and Apela in the migration of prospective endothelial cells during vasculogenesis, each separately in different organisms. Together, our results suggest that, in mice, VEGF and Apela may both play a role in the migration of cells that results in the formation of the endocardium, dorsal aortae, and head vasculature.

### Cells are budding from the wall of the endocardium

**Fig. 6:**
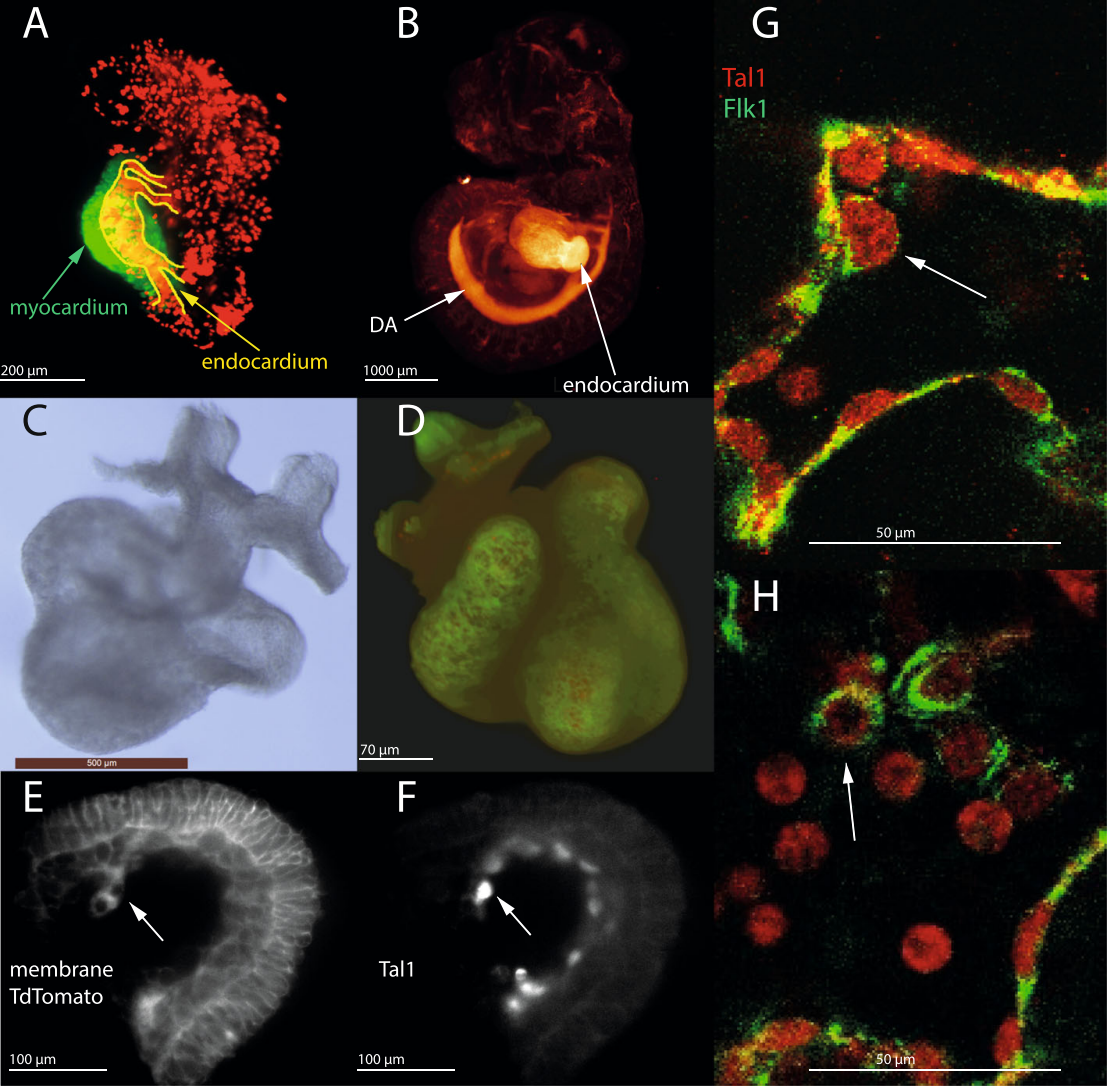
Cells are budding from the endocardium. (**a, b**): stills from Additional File 33 and Additional File 34 of Tal1 expression in E8.5 (non-cleared) and E10.5 (cleared) embryos, imaged by light sheet microscopy and by OPT respectively, showing strong expression of Tal1 in the endocardium. (**c**): brightfield image of E9.5 heart. (**d**): the heart shown in (**c**) expressing membrane tdTomato and Tal1-cerulean. (**e,f**): light-sheet images of calls budding from the wall of the endocardium. (**e**): membrane tdTomato expression. (**f**): Tal1 expression. (**g,h**): Tal1 and Flk1 expression in cells in the endocardium of E9.5 hearts, imaged by confocal microscopy

We have shown that some or all of the prospective endothelial cells that form the endocardium, the dorsal aortae and the vasculature in the head are derived from the same pool of cells in the distal border of the yolk sac, and it is known that secondary haematopoiesis takes place in the dorsal aortae and head vasculature, so it is interesting to ask whether it also occurs in the endocardium. Tal1 is expressed in haemogenic endothelial cells, and when levels of Tal1 reach a certain threshold, such cells become irreversibly committed to haematopoiesis [12]. Light sheet microscopy of an E8.5 embryo, and optical projection tomography of an E10.5 embryo, reveal that Tal1 is strongly expressed in the endocardium during later stages of embryonic development (https://youtu.be/rKP0QA0xB0Y, Additional File 33; https://youtu.be/fT2VQ1al8LM, Additional File 34; Fig. S1M; and Fig. 6a, b).

Using a mouse line that expresses Tal1-cerulean and membrane-targeted TdTomato, to visualise the cell surface as well as the nucleus, we discovered cells budding from the endocardium of an E9-E9.5 mouse heart (Fig. 6c, d, e, f). To confirm this finding, we carried out immunostainings of dissected E9.5 hearts with Flk1 and Tal1 antibodies. Cells within the endocardium were Flk1 and Tal1 positive, and we observed endothelial cells detaching from the wall of the endocardium in which expression of Flk1 was substantially reduced (Fig. 6g, h). Haematopoietic cells lose Flk1 expression [27] and the cell shape of the detached cells was significantly rounder (Fig. 6h). The exact nature of these cells remains to be determined, but together, our results indicate that an endothelial to haematopoietic transition is taking place.

## Discussion

Understanding the spatial origin and migration pathways of cells as they differentiate is an essential aspect of development, but open questions remain for the initial formation of the vasculature in mouse. Classic embryology concluded that there are two independent distinct processes—extra and intra-embryonic vasculogenesis—suggesting that the angioblasts that form them are induced in situ by different mechanisms, and this view persists. In contrast, we show that the intra- and extra-embryonic blood vessels are, at least in part, formed by a common pool of cells, and that migration from the distal margin of the yolk sac is involved.

**Fig. 7:**
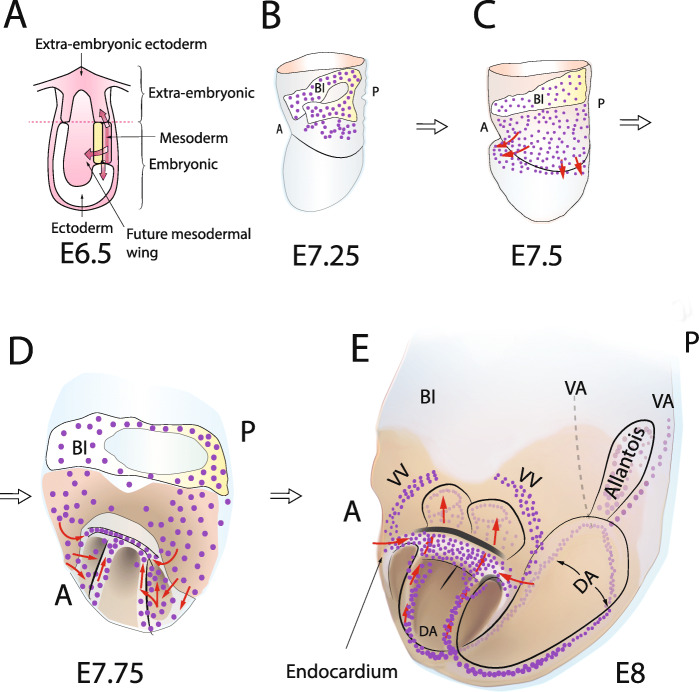
Formation of the primary circulatory system, overview. A = anterior, P = posterior, BI = blood islands, DA = dorsal aortae, VV = vitelline veins, VA = vitelline arteries. (**a**): section through an E6.5 embryo (as in [28]), showing that cells of the epiblast, at the border of the extra-embryonic region and the epiblast, become mesoderm and form the primitive streak. Cells at the top of the primitive streak migrate upwards into the extra-embryonic region and the primitive streak expands downwards into the embryonic region. Mesodermal cells also migrate all around, just underneath the outer (endoderm) layer, in the embryonic and extra-embryonic regions; the former become the mesodermal wings (MW). (**b**): between E7 and E7.25, a fraction of the mesodermal cells in the extra-embryonic region acquires haemato-endothelial cell fate in the region where the BI are developing and start expressing Tal1. (**c**): at E7.5, Tal1 positive cells spread throughout the YS and the bud of the allantois (interior, not shown), and are also seen in the embryo near the anterior part of the neural tube. They may be attracted by a chemo-attractant secreted at the anterior part of the midline, and repulsed by a signal with a more limited range, coming from the notochord, leaving the observed symmetrical Tal1 positive-cell-free zone on either side of the midline. (**d**): at E7.75, cells have also migrated out of the yolk sac into the middle and posterior part of the embryo. Again, cells arrest at a distance from the midline on either side. The cells that previously migrated towards the anterior part of the neural tube are now localised just above the anterior intestinal portal, where they start forming the endocardium, while the head folds (interior, not shown) are developing towards the inside of the embryo. Some cells have started to ingress into the head folds. (**e**): at E8, cords of endothelial cells start forming the dorsal aortae at either side of the midline, where they stopped migrating. At the anterior part of the embryo, the dorsal aortae extend into the head folds and cells move from the dorsal aortae underneath the endocardium straight into the head folds. Cells from the yolk sac keep contributing to the endocardium, which is now a pronounced crescent. In the yolk sac, the omphalomesenteric system develops, connecting the back of the dorsal aortae with the blood islands via the vitelline arteries at the posterior part of the embryo, and the venous poles of the heart with the blood islands via the vitelline veins at the anterior part of the embryo. Tal1 positive cells remain in the rest of the yolk sac and the blood islands but have been omitted here for clarity

In Fig. 7 we summarise the initial formation of the vasculature and highlight the cell migration that we observe. As outlined in [28] and shown in [29] and, with live imaging, in [30], at E6.5 mesoderm cells from the top of the primitive streak migrate into the extra embryonic domain (Fig. 7a). At E7.0–7.25, cells in the region of the forming blood islands acquire a haemato-endothelial fate (Fig. 7b) and start expressing Tal1. At E7.5, we see Tal1 positive cells in a speckled pattern uniformly across most of the yolk sac. From E7.5 our live imaging shows Tal1 positive cells leaving the yolk sac in the anterior region of the embryo, to contribute to the endocardium (Fig. 7c). In the middle and posterior regions, we see Tal1 positive cells leave the distal border of the yolk sac to contribute to the dorsal aortae on either side of the midline (Fig. 7d). At the anterior part of the embryo, we see cells from the dorsal aortae migrate underneath the endocardium directly into the headfolds, to contribute to the vasculature of the head (Fig. 7e). At E7.75, vitelline arteries develop in the posterior part of the yolk sac and connect the dorsal aortae to the blood islands (Fig. 7e). At E8 the vitelline veins start forming in the yolk sac at the anterior part of the embryo to connect the venous poles of the endocardium to the blood islands (Fig. 7e), completing the formation of the primary circulatory system.

In our imaging data, the Tal1 positive cells that contribute to this embryonic and extra-embryonic initial vasculature all arise from the apparently uniform speckled pattern of Tal1 positive cells across the yolk sac at E7.5, with no distinction between them except their location and the later observed migration of (at least some of) those that form the embryonic vasculature. This Tal1 speckled pattern cannot be seen in the blood island region, where there is bright fluorescence from the blood progenitors, but the Flk1 data show a similar speckled pattern uniformly across the entire yolk sac, so it may be that the Tal1 signal is the superposition of such a pattern and the blood island expression. We do not see large-scale migration within the bulk of the yolk sac, or from the blood islands.

Given this distribution and migration pattern of the cells that form the initial vasculature, we look at the signals that might control it. Our results are consistent with Tal1 positive cells being first attracted towards the anterior part of the neural tube and the pharyngeal mesoderm and subsequently towards the rest of the midline of the embryo. We see that Tal1 positive cells are able to migrate on top of the anterior neural tube and that later, the cells that form the outflow tract of the endocardium form a semicircle around the headfolds, consistent with a chemo-attractant expressed in the headfolds. We show that VEGF and Apela are strong candidate chemo-attractants that might direct the migration of the cells. Chemo-attractants are not the only signals involved in shaping the initial vasculature: others have shown that this requires multiple redundant repulsive guidance cues. Expression of these in the notochord causes the avascular zone that first develops at the midline, and Semaphorin 3E, a strong repellent of endothelial cells, has been shown to be required for the formation of the two avascular regions in the lateral plate mesoderm [16]. From our temporal and spatial expression pattern of Tal1 positive cells, we can conclude that cells first migrate from the distal end of the yolk sac towards the midline of the embryo to form the vasculature, moving over the later avascular region, after which the lateral plate mesoderm is cleared of Tal1 positive cells. Future experiments showing the temporal and spatial expression patterns of chemo-attractants and repellents superimposed on the expression pattern of Tal1 will help us understand the formation of the initial vasculature in greater detail.

Finally we noticed cells budding from the wall of the endocardium at E9.5 with high Tal1 expression and diminished Flk1 expression and with a round appearance, both signs of endothelial to haematopoietic transition (see [27] for the former). We did not investigate the specific cell type of the budding cells, but our results are consistent with those of Nakano et al. [31], who discovered that a subset of endocardial endothelial cells gives rise to erythroid/myeloid progenitors at E8.25-E9.5. This is in contrast to the observations of Lux et al. [32], who describe that secondary haematopoiesis takes place exclusively in the yolk sac.

## Conclusion

We show that Tal1 positive cells migrate from the distal end of the yolk sac to contribute to the endocardium, dorsal aortae and vasculature of the head; the same pool of cells also contributes to the vasculature in the yolk sac, including the vitelline arteries. This contradicts the notion that the intra-and extra-embryonic vasculogenesis are separate and independent processes. We identify VEGF and Apela as potential chemo-attractants involved in this process.

Between E10.5 and E11.5, haematopoietic stem cells have been shown to arise independently in the dorsal aortae [33] and in the head [34], and cells that can be cultured to become haematopoietic stem cells are found in the arteries of the umbilical cord [35]. As we have shown that the vasculature in these three regions contains cells derived from the same pool in the distal end of the yolk sac, it will be interesting for future studies to determine whether these haematopoietic stem cells have a common progenitor there.

Moreover, chemo-attractants have been shown to also affect cell specification, e.g. VEGF is used in protocols to generate haematopoietic stem cells in vitro [36]. The other signals involved in controlling the migration paths of these cells, including Apela, may also have a role here.

## Methods

### Mouse strains used in this study

Tal1 is encoded on Chromosome 4: 115,056,426-115,071,755 (MGI:98480). We designed a construct containing a Tal1 homology arm overlapping with the 3′ end of the Tal1 ORF, followed by a T2A sequence encoding a self-cleaving peptide, a cerulean sequence [37] with N-terminal HIST1H2BJ tag for nuclear localisation of the fluorophore, and a Tal1 homology arm in the 3′ UTR of Tal1 (full sequence attached in Additional File 35). Single-stranded DNA was ordered from Integrated DNA Technologies (IDT). Founder mice were obtained by pronuclear injection of this DNA, together with Cas9 protein (IDT) and guide RNA (final concentrations were 20 ng/ul for each component), into zygotes of the C57BL/6 J strain [38]. 275 zygotes were transferred, resulting in the birth of 32 offspring, 15 of which were positive for the insert. Nine founder mice were shown to have the correct sequence by PCR and Miseq. Following long-range PCR, further sequencing was able to verify most of the insert sequence (all relevant DNA was fully sequenced except a 219 bp area of the cerulean region). Three founder mice were then bred. All the validation steps were repeated on the F1 offspring of two founder lines, 3.1a and 1.1 g, and these were maintained for further study. All mice used in this study were made homozygous for Tal1-cerulean, because this significantly improved the strength of the fluorescent signal.

The Flk1-GFP line was obtained from the JAX repository and made by [6].

The Tal1-cerulean line was crossed with a line expressing Cre recombinase in the myocardium [39] and the resulting line was crossed with the Rosa26Rtdtomato line [40], to visualize endocardium as well as myocardium. The Tal1-cerulean line was also crossed with the Rosa26-mTmG C57BL6j [41] line, to visualize endocardial cells as well as the cell membrane of every cell.

### Immunostaining

The immunostaining protocol described in [42] was modified as follows: First, embryos were dissected in FHM medium (described in supplement). They were fixed in PBS with 4% PFA, 0.1% Tween and 0.01% Triton for 15 min at room temperature (RT). They were then washed twice for 5 min in PBS and permeabilized with 0.5% Triton in PBS for 20 min at RT. Embryos were briefly rinsed with PBS with 0.1% Triton and further washed for 3 × 5 min at room temperature (RT). Blocking solution, consisting of 5% donkey serum (Millipore Cat# S30–100 ml) in PBS with 0.1% Triton and 2% BSA was added to the embryos for 48 h at 4 °C. The embryos were incubated overnight in primary antibody diluted in blocking solution at 4 °C. They were then washed in PBS with 0.1% Triton, the first time briefly, then for 6 × 30 min at room temperature. The secondary antibody, diluted in blocking solution, was added to the embryos for 2 h in the dark at 4 °C. Embryos were rinsed in PBS with 0.1% Triton and washed for 15 min in PBS with 0.1% Triton and 0.5 μg/ml DAPI. They were then washed for 6 × 30 min in PBS with 0.1% Triton. Embryos were mounted in 0.2% agarose in PBS in glass bottom MatTek dishes (Cat# P35G-1.5-20-C) and imaged with a Zeiss LSM 710 confocal microscope.

### Antibodies

Rabbit anti-human Tal1 antibody [C2C3] C-term from GeneTex (1 mg/ml) (Cat# GTX116020) was used at 1/100. Rat anti-mouse Flk1 antibody from BD Pharmingen (0.5 mg/ml) (Cat# 555307) was used at 1/100. Secondary antibodies Alexa Fluor 594 donkey anti-rabbit IgG (2 mg/ml) (Cat# A21207) and Alexa Fluor 488 donkey anti-rat IgG (2 mg/ml) (Cat# A21208) were used at 1/500.

### Microscopes used in this study

These included MuVi SPIM (Bruker-Luxendo) and UltraMicroscope Series 2 (Miltenyl-LaVision BioTec) light sheet microscopes; an optical projection tomography (OPT) microscope (built in situ as described below); TCS SP5 (Leica); LSM 710 confocal (Zeiss).

### Dissection, fixation, clearing, and mounting of embryos for imaging

Embryos were dissected in FHM medium (95 mM NaCl; 2.5 mM KCl; 0.35 mM KH_2_PO_4_; 0.2 mM MgSO_4_.7H2O; 10 mM Na lactate; 0.2 mM Na pyruvate; 0.2 mM Glucose; 1.0 mM Glutamine; 0.01 mM EDTA; 4.0 mM NaHCO_3_; 1.71 mM CaCl_2_.2H_2_O; 20 mM HEPES; pH adjusted to pH 7.4; after which 1 g/L BSA and 1x Pen/strep (Cat# GIBCO 15140148) were added).

For imaging with the light sheet MuVi SPIM, embryos were fixed in PBS with 4% PFA (ThermoScientific, Cat# 28908) for 10 min at room temperature (short fixation time to reduce background), mounted in 2% LMP agarose (Thermo Scientific, Cat# R0801) in PBS, and imaged immediately.

For imaging with the light sheet UltraMicroscope or OPT, embryos were fixed in PBS with 4% PFA overnight at 4 °C. They were cleared in UBAS-M (25 wt% Meglumine (Sigma-Aldrich, Cat# M9179), 25 wt% Urea (Sigma-Aldrich, Cat# 15604), 20 wt% 1,3-Dimethyl-2-imidazolidinone (Sigma-Aldrich, Cat# 40727), and 0.2 wt% Triton X-100 (Sigma-Aldrich, Cat# 10789704001)) [43] in 50 ml per embryo overnight at RT and mounted in 4% LMP agarose in UBAS-M before imaging.

### Dissection, mounting, and culturing of embryos for live imaging

Embryos were dissected in DMEM/F-12 (GIBCO Cat# 11039–021) with 1x Glutamax (GIBCO Cat # 3505–038); 10% FBS (Thermo Fisher Cat# 10270106) and 1x Penicillin/Streptomycin (Pen/Strep) (Cat# GIBCO 15140148). They were cultured in rat serum from female rats (Charles River) that had been filtered through a 0.2 μm Minisart Syringe Filter (Sartorius Cat# 16534), denatured at 56 °C for 30 min and to which 1x Pen/Strep had been added.

For live imaging with the MuVi SPIM, embryos were mounted in custom-made coils of 0.1 mm titanium wire (shown in Fig. S2–4) and inserted in FEP tubing (inner diameter 1.7 mm) filled with rat serum. For live imaging with the TCS SP5 two-photon microscope, embryos were mounted in drilled or laser-cut acrylic holders that were placed in glass-bottom MatTek dishes (P35G-1.5-14-C) in rat serum covered with mineral oil, as in [44]). Live imaging was carried out in customised heated chambers at 37 °C in a 5% O_2_, 5% CO_2_, 90% N_2_ atmosphere.

### Imaging and image processing of fixed embryos with the MuVi SPIM

The MuVi SPIM is equipped with two sCMOS Orca Flash 4.0 v3 cameras (Hamamatsu), two Nikon CFI Plan Fluor 10x W NA 0.3 objectives for illumination and two Olympus XLUMPLFLN 20x W NA 1.0 objectives for signal detection. All images were acquired using a tube lens to yield an effective magnification of 16.6x. Tal1-cerulean embryos were illuminated with a 445 nm laser and a 457–501 nm bandpass emission filter was used. Distance between planes in the z-stack was 2 μm and light sheet thickness was 4.1 μm. The field of view was 803 × 803 μm (2048 × 2048 pixels). Flk1-GFP embryos were imaged with a 488 nm laser and a long-pass 498 emission filter and the same volume parameters as used for Tal1-cerulean embryos.

Embryos were imaged at 4 different angles (0°, 45°, 90°, 135°) and, when larger than the field of view, with tiling. Images obtained by the left and right cameras were fused using the Luxendo GUI. Images from different rotational views and tiles were converted to TIFF using the using the BigDataProcessor plugin (https://zenodo.org/record/2575681#.X89wUdiwl3g), and fused using the BigStitcher plugin (https://rdcu.be/b3enW) from FIJI [45]. Embryos were visualised and cropped in 3D in Imaris to eliminate visible debris in the surrounding medium. Movies were created and overlays were added to the movies in FIJI.

### Imaging and image processing of fixed embryos with the LaVision UltraMicroscope light sheet

Embryos were imaged with the light sheet Ultramicroscope Series 2 from Miltenyl-LaVision BioTec, equipped with an Andor Zyla camera (2560 × 2560 pixels) and MVPLAPO 2X NA 0.5 detection lens. For the acquisition we used a 488 nm laser and an emission bandpass filter at 525–550 nm. Light sheet thickness was 40 μm and zoom factor 0.8, providing a final magnification of 1.7x. Acquisition was performed using double-side illumination and blending algorithm to fuse the images. For each volume, the z-step size was 5 μm and field of view 8.2 × 9.7 mm. Visualization of the embryos and movie making were done in Imaris. Overlays were added to the movies in FIJI.

### Imaging and image processing of fixed embryos by OPT

Embryos were imaged with an Optical Projection Tomography (OPT) system with both a 1x and a 2x imaging arm. The 1x arm comprises a telecentric lens (#58–430 TechSpec SilverTL, Edumund Optics) with adjustable aperture stop, with a dielectric filter placed in front for wavelength selection. The 2x arm comprises a microscope objective (2x CFI Plan Apochromat, Nikon) and tube lens (TTL-200A, Thorlabs), with an adjustable aperture placed behind the microscope objective to allow for varying the system numerical aperture whilst maintaining object-space telecentricity, and a filter placed in front of the camera for emission wavelength selection. In all cases, the camera used is an sCMOS sensor (Zyla 5.5, Andor). Illumination is provided by a multiline laser light source (TriLine, Cairn), with 460,555 and 635 nm lines available), which is coupled through an actively vibrated 800 μm core diameter fibre in order to homogenise the beam profile. The beam is collimated, passed through an excitation filter, then focused onto a diffuser for further homogenisation, following which a Fresnel lens (FRP251, Thorlabs) is used to collimate the light coming off the diffuser and to illuminate the sample; in the case of smaller samples, this lens can be moved further from the diffuser to partially focus the illumination on a smaller sample region. The excitation (FF01–445/45–25) and emission filters (FF01–500/24–25) used for mTurquoise imaging are both from Semrock.

Images were registered and reconstructed using custom filtered backprojection software written in MATLAB to run on a GPU to produce 3D image stacks, then visualised with Imaris, and a legend added with FIJI.

### Imaging and image processing of live embryos with the MuVi SPIM

Tal1-cerulean embryos were imaged at a single angle with a 445 nm laser and with bandpass 457–501 nm emission filter. Volumes (z-step 2 μm, field of view 803 × 803 μm) were acquired every 11 min using a light sheet thickness of 4.1 μm. While the embryo was growing, the region of interest was kept in the field of view by moving the stage. Images obtained by the left and right cameras were converted to HDF5 file format with the Luxendo GUI, saved to TIFF using the BigDataProcessor plugin (https://zenodo.org/record/2575681#.X89wUdiwl3g), and fused with the BigStitcher plugin (https://rdcu.be/b3enW). Time series with different stage coordinates were registered and fused using FIJI. Images were visualised in Imaris and embryos were 3D cropped to eliminate visible debris in the medium.

### Imaging and image processing of live embryos with the TCS SP5 Leica microscope

Embryos were imaged with the TCS SP5 microscope from Leica with hybrid detectors, and.

using the 20 × 1.0 W lens from Leica (HC x APO). Tal1-cerulean embryos were imaged taking a stack every 10′45″. The step size in Z was 6 μm. Flk1-GFP embryos were imaged taking a stack was every 10′ 35″. The step size in Z was 9 μm. The field of view was 760 μm × 760 μm. Data were processed in FIJI. A maximum projection of the fluorescent signal was obtained and the sharpest brightfield image of each stack was selected by the stack focuser plugin from FIJI. Both channels were merged and overlays were added in FIJI.

### Single cell transcriptomics

We followed the protocol described in [46] with minor modifications. Embryos were dissected in FHM medium (as described above). They were transferred onto a dish covered with a layer of 1% agarose in HBSS medium (Thermofisher Cat# 88284) and filled with HBSS with 5% Foetal Bovine Serum (FBS). The ‘top’ and ‘bottom’ part of 25 embryos (E7.25-E7.5) were sliced off, using a 13-Y1 micron wire tip cautery electrode with 1 mm loop from πProtech, mounted on a Model MC-2010 micro cautery instrument. ‘Top’ and ‘bottom’ parts were separated immediately after cutting and stored in Low-bind Eppendorf tubes (Cat# 022431021) filled with 500 μl recovery medium (HBSS with 5% FBS, rock inhibitor (10 μm, Sigma Aldrich Y-27632) and 1x non-essential amino acids (Thermofisher Cat# 11140050)) at 37 °C, until all pieces sank to the bottom of the tube. The recovery medium was removed by pipetting and 500 μl of dissociation medium (FACSmax cell dissociation solution (Amsbio T200100) with 10x papain (Sigma Aldrich Cat# 10108014001)) was added at 37 °C for a total time of 10′. Every 2′, cells were pipetted up and down carefully with 200 μl MultiGuard Barrier Tips, Sorenson Cat#30550 T. Cells were then pipetted through a cell restrainer (pluriSelect, pluristrainer 15 μm Cat# 43–50,015-01), using a 1000 μl non-stick filter tip. Cells were spun down for 2′ at 1300 rpm, the dissociation medium was removed and cells were re-dissociated in 500 μl recovery medium (see above). Cells were spun down for 2′ at 1300 rpm for a second time and re-dissociated in recovery medium.

Cell size, number of cell aggregates, and percentage of live cells were used as measures of quality control, and only samples with a viability over 65% were used. A suspension of 10,000 single cells was loaded onto the 10x Genomics Single Cell 3′ Chip, and cDNA synthesis and library construction were performed as per the manufacturer’s protocol for the Chromium Single Cell 3′ v2 protocol (10x Genomics; PN-120233). cDNA amplification involved 12 PCR cycles).

### Single cell transcriptomics data analysis

Cells of ‘top’ and ‘bottom’ samples were clustered according to the manufacturer’s methods (https://support.10xgenomics.com/single-cell-gene-expression/software/pipelines/latest/algorithms/overview), following the Graph-based section. Supplemental Table 3 was generated following the Differential expression-based section.

## Supplementary Information

**Additional file 1.** Brightfield images of the embryos in the movies. A: Additional File 2. B: Additional File 3 and 4; C: Additional File 6 and 7; D: Additional File 8 and 9; E: Additional File 10; F: Additional File 12; G: Additional File 22; H: Additional File 23; I: Additional File 24; J: Additional File 25; K: Additional File 26; L: Additional File 27; M: Additional File 34.

**Additional file 2.** Tal1 expression in an E7.25 embryo rotating around its proximal distal axis. A = anterior, P = posterior, BI = blood islands. Tal1 expression in an E7.25, fixed, non-cleared embryo, imaged by light sheet microscopy, rotating around the vertical axis of the video.

**Additional file 3.** Tal1 expression in an E7.5 embryo rotating around its proximal distal axis. A = anterior, P = posterior, YS = yolk sac, ML = midline, BI = blood islands, ECs = prospective endothelial cells. Tal1 expression in an E7.5, fixed, non-cleared embryo, imaged by light sheet microscopy, rotating around the vertical axis.

**Additional file 4.** Tal1 expression in an E7.5 embryo rotating around its lateral axis A = anterior, ML = midline, ECs = prospective endothelial cells, BI = blood islands, YS = yolk sac. Tal1 expression in an E7.5, fixed, non-cleared embryo, imaged by light sheet microscopy, rotating around the horizontal axis.

**Additional file 5.** Tal1 expression in the anterior part of an E7.5 embryo A = anterior, ML = midline, BI = blood islands, YS = yolk sac. Tal1 expression in the dissected anterior part of an E7.5, fixed, non-cleared embryo, imaged by light sheet microscopy, rotating around the horizontal axis.

**Additional file 6.** Tal1 expression in an E7.75 embryo rotating around its proximal distal axis A = anterior, P = posterior, YS = yolk sac, ECs = endothelial cells. Tal1 expression in an E7.75, fixed, non-cleared embryo, imaged by light sheet microscopy, rotating around the vertical axis.

**Additional file 7.** Tal1 expression in an E7.75 embryo rotating around its lateral axis. A = anterior, ML = midline, YS = yolk sac. Tal1 expression in an E7.75, fixed, non-cleared embryo, imaged by light sheet microscopy, rotating around the horizontal axis.

**Additional file 8.** Tal1 expression in an E8 embryo rotating around its proximal distal axis A = anterior, P = posterior, YS = yolk sac, BI = blood islands, HF = head folds, DA = dorsal aortae. Tal1 expression in an E8, fixed, non-cleared embryo, imaged by light sheet microscopy, and rotating around the vertical axis.

**Additional file 9.** Tal1 expression in an E8 embryo rotating around its lateral axis A = anterior, ML = midline, HF = head folds, VA = vitelline arteries. Tal1 expression in an E8, fixed, non-cleared embryo, imaged by light sheet microscopy, and rotating around the horizontal axis.

**Additional file 10.** Tal1 expression in the anterior part of an E8 embryo A = anterior, DA = dorsal aortae, HF = head folds. Tal1 expression in the dissected anterior part of an E8, fixed, non-cleared embryo, imaged by light sheet microscopy, and rotating around the horizontal axis.

**Additional file 11.** Tal1 expression in the anterior part of an E8 embryo A = anterior, DA = dorsal aortae, HF = head folds. Tal1 expression in the dissected anterior part of an E8, fixed, non-cleared embryo, imaged by light sheet microscopy, and rotating around the horizontal axis.

**Additional file 12.** Tal1 expression in an E9.5 embryo A = anterior, DA = dorsal aortae. Tal1 expression in an E9.5, fixed, cleared embryo, dissected to remove tail, imaged by OPT, and rotating around the vertical axis.

**Additional file 13 **Mounting an embryo for live imaging with the light sheet microscope The MuVi SPIM light sheet microscope stage holds a glass capillary; a piece of transparent FEP tube (that does not optically interfere with imaging) slides over top of the glass tube and provides a watertight seal: the sample must be held within this. For live imaging of embryos, we developed a new mounting protocol. Embryos were held by their extra-embryonic cones in a single coil of 0.1 mm titanium wire (to minimise toxicity). (**A**) A large coil was made at one end of a piece of 0.1 mm titanium wire, by winding the wire around the shank of a 0.7 mm drill bit, clamped in a vice. This large coil is designed to anchor the wire into agarose, and to centre the smaller embryo-holding coil. (**B, C**) To make the embryo-holding coil, the large coil is pulled over the shank of another drill bit, with a diameter suited to the size of the embryo that needs to be mounted (usually between 0.15 and 0.45 mm) and a single coil is made by winding the wire (trimming the excess). (**D**) Glass capillary with diameter 1.6 mm, and insulated wire (blue) that fits into the glass tube. (**E**) The blue wire is inserted into the glass capillary. (**F**) By pulling the end of the blue wire out of the glass capillary, while holding the other end of the glass capillary in 2% molten LMP agarose in CMERL-1066 medium (PANBiotech Cat# P04–84600), agarose is sucked into the glass capillary.

**Additional file 14 **Mounting an embryo for live imaging with the light sheet microscope (**A**) The titanium wire is pushed through the glass capillary filled with molten agarose, leaving a small coil sticking out at one end and the bottom of the wire at the other. The blue wire is partially inserted next to the titanium wire. Both wires together will act as a plunger, once the agarose has set, to be able to move the small coil. (**B**) A piece of FEP tube is filled with rat serum and slid partially over the end of the glass tube. (**C**) The tip of the FEP tube is immersed in a droplet of rat serum that has been pipetted onto a pre-warmed, water-repellent PTFE plate (to keep the serum as a convenient droplet). The plunger is used to push the tip of the small coil through the FEP tube into the droplet. (**D**) The embryo is mounted in the top coil inside the droplet, holding it by the detached Reichert’s membrane. (**E**) The plunger is pulled back slowly, moving the embryo into the FEP capillary filled with rat serum, and the excess titanium and blue wire is trimmed. (**F**) The embryo is positioned just above the glass tube in the FEP tube.

**Additional file 15 **Mounting an embryo for live imaging with the light sheet microscope (**A**) The entire contraption is mounted into the pre-warmed, water-filled chamber (with custom-made heater) of the MuVi SPIM microscope, leaving the top of the FEP tube sticking out above the water level. (**B**) The chamber is closed with a lid that is connected to a gas tank to create the right atmosphere (see Materials and Methods).

**Additional file 16.** Tal1 expression in a live embryo rotating around its proximal distal axis A = anterior, P = posterior. Tal1 expression in a live E7.5 embryo, imaged for 9 h. 43 min. by light sheet microscopy, and rotating 180° around the vertical axis.

**Additional file 17.** Manual cell tracking in a live Tal1 expressing embryo A = anterior, P = posterior, DA = dorsal aortae. Tal1 expression and cell tracks of cells migrating from the yolk sac to the dorsal aortae in a live E7.5 embryo, imaged for 9 h. 43 min. by light sheet microscopy.

**Additional file 18.** Manual cell tracking in a live Tal1 expressing embryo A = anterior, P = posterior, DA = dorsal aortae. Tal1 expression and cell tracks of cells in the embryo returning to the yolk sac, and of a cell remaining in the yolk sac, in a live E7.5 embryo, imaged for 9 h. 43 min. by light sheet microscopy.

**Additional file 19.** Manual cell tracking in a live Tal1 expressing embryo A = anterior, P = posterior, DA = dorsal aortae. Tal1 expression and cell tracks of cells migrating from the yolk sac to the endocardium in a live E7.5 embryo, imaged for 9 h. 43 min. by light sheet microscopy.

**Additional file 20.** Automated cell tracking in a live Tal1 expressing embryo A = anterior, P = posterior, DA = dorsal aortae. Tal1 expression and cell tracks of all Tal1 positive cells in a live E7.5 embryo, imaged for 9 h. 43 min. by light sheet microscopy.

**Additional file 21.** Tal1 expression in the anterior part of a live embryo A = anterior. Tal1 expression in a live E 7.75 embryo, imaged for 8 h by two-photon microscopy. Cell flow in the dorsal aortae, and the cell flow of cells that contribute to the endocardium, are indicated by yellow arrows. A sample of cells migrating on top of the neural tube are circled in yellow, as are dividing cells where one daughter goes to the dorsal aortae and the other to the endocardium.

**Additional file 22.** Flk1 expression in an E7 embryo rotating around its proximal distal axis A = anterior, P = posterior, MW = mesodermal wing. Flk1 expression in an E7, fixed, non-cleared embryo, imaged by light sheet microscopy, and rotating around the vertical axis.

**Additional file 23.** Flk1 expression in an E7.5 embryo rotating around its proximal distal axis A = anterior, P = posterior, ECs = endothelial cells, YS = yolk sac. Flk1 expression in an E7.5, fixed, non-cleared embryo, imaged by light sheet microscopy, and rotating around the vertical axis.

**Additional file 24.** Flk1 expression in an E7.75 embryo rotating around its proximal distal axis A = anterior, P = posterior, DA = dorsal aortae, YS = yolk sac, ECs = endothelial cells. Flk1 expression in an E7.75, fixed, non-cleared embryo, imaged by light sheet microscopy, and rotating around the vertical axis.

**Additional file 25.** Flk1 expression in an E8 embryo rotating around its proximal distal axis A = anterior, P = posterior, DA = dorsal aortae, HF = head folds, VV = vitelline veins, YS = yolk sac. Flk1 expression in an E8, fixed, non-cleared embryo, imaged by light sheet microscopy, and rotating around the vertical axis.

**Additional file 26.** Flk1 expression in an E9 embryo A = anterior, DA = dorsal aortae, ISBV = inter-somitic blood vessels. Flk1 expression in an E9, fixed, cleared embryo, imaged by light sheet microscopy.

**Additional file 27.** Flk1 expression in an E9.5 embryo A = anterior, DA = dorsal aortae. Flk1 expression in an E9.5, fixed, cleared embryo, imaged by light sheet microscopy.

**Additional file 28.** Flk1 expression in a live embryo A = anterior, P = posterior. Flk1 expression in a live E7 embryo, imaged for 14 h by two-photon microscopy.

**Additional file 29 **Clusters of top and bottom samples (**A, B**) t-distributed stochastic neighbour embedding (t-SNE) plots, showing clusters of cells of the ‘top’ part (as shown in Fig. 4a) of twenty-five (E7.25-E7.5) embryos, and the localisation of those clusters in the aggregate (**B**). The Tal1 expressing cells are those of cluster 5, and the Sox17 expressing cells are those of clusters 1 and 6. (**C, D**) t-SNE plots, showing clusters of cells of the ‘bottom’ part of twenty-five (E7.25-E7.5) embryos, and the localisation of those clusters in the aggregate (**D**). The Tal1 expressing cells are those of cluster 8 and the Sox17 expressing cells are those of cluster 6. Highly expressed genes in the different clusters can be found in Table S.1 and Table S.2.

**Additional file 30 **Genes expressed in the clusters of the top sample Genes expressed in clusters 1–6 of the ‘top’ sample. Indicated per cluster are the mean of the UMI counts per gene, the Log_2_ of the fold change of the average number of reads for a given gene across cells in that cluster to that of cells not in the cluster, and the significance (*p*-value) of the Log_2_-fold change.

**Additional file 31.** Genes expressed in the clusters of the bottom sample Genes expressed in clusters 1–8 of the ‘bottom’ sample. Indicated per cluster are the mean of the UMI counts per gene, the Log_2_ fold change of the average number of reads for a given gene across cells in that cluster to that of cells not in that cluster, and the significance (p-value) of the Log_2_-fold change.

**Additional file 32.** Genes differentially expressed in the endoderm of top versus bottom sample Genes differentially expressed in endoderm of ‘top’ versus ‘bottom’ sample. A positive Log_2_-fold change indicates that the gene is upregulated in the ‘top’ sample versus the ‘bottom’ sample. A negative Log_2_-fold change indicates the converse.

**Additional file 33.** The endocardium and myocardium in an E8.5 embryo A = anterior, P = posterior. Tal1 expressed in endocardium, dorsal aortae, and head vasculature in red; tdTomato expression in myocardium in green, in an E 8.5 fixed, non-cleared embryo, imaged by light sheet microscopy and rotating around the vertical axis.

**Additional file 34.** Tal1 expression in an E10.5 embryo A = anterior, DA = dorsal aortae. Tal1 expression in endocardium and dorsal aortae in an E 10.5 fixed, cleared embryo, imaged by OPT and rotating around the vertical axis.

**Additional file 35.** Map of the Tal1-cerulean construct Map and full sequence of the Tal1-cerulean construct, inserted in exon 5 of the Tal1 genomic region.

## Data Availability

Raw data for the single cell transcriptomics experiment are available on the GEO platform as series GSE165402.
