# Supplementary figures and images for: The migratory pathways of the cells that form the endocardium, dorsal aortae, and head vasculature in the mouse embryo
Source: BMC Dev Biol. 2021 Mar 22;21:8. doi: 10.1186/s12861-021-00239-3 (PMC7986287; doi:10.1186/s12861-021-00239-3)

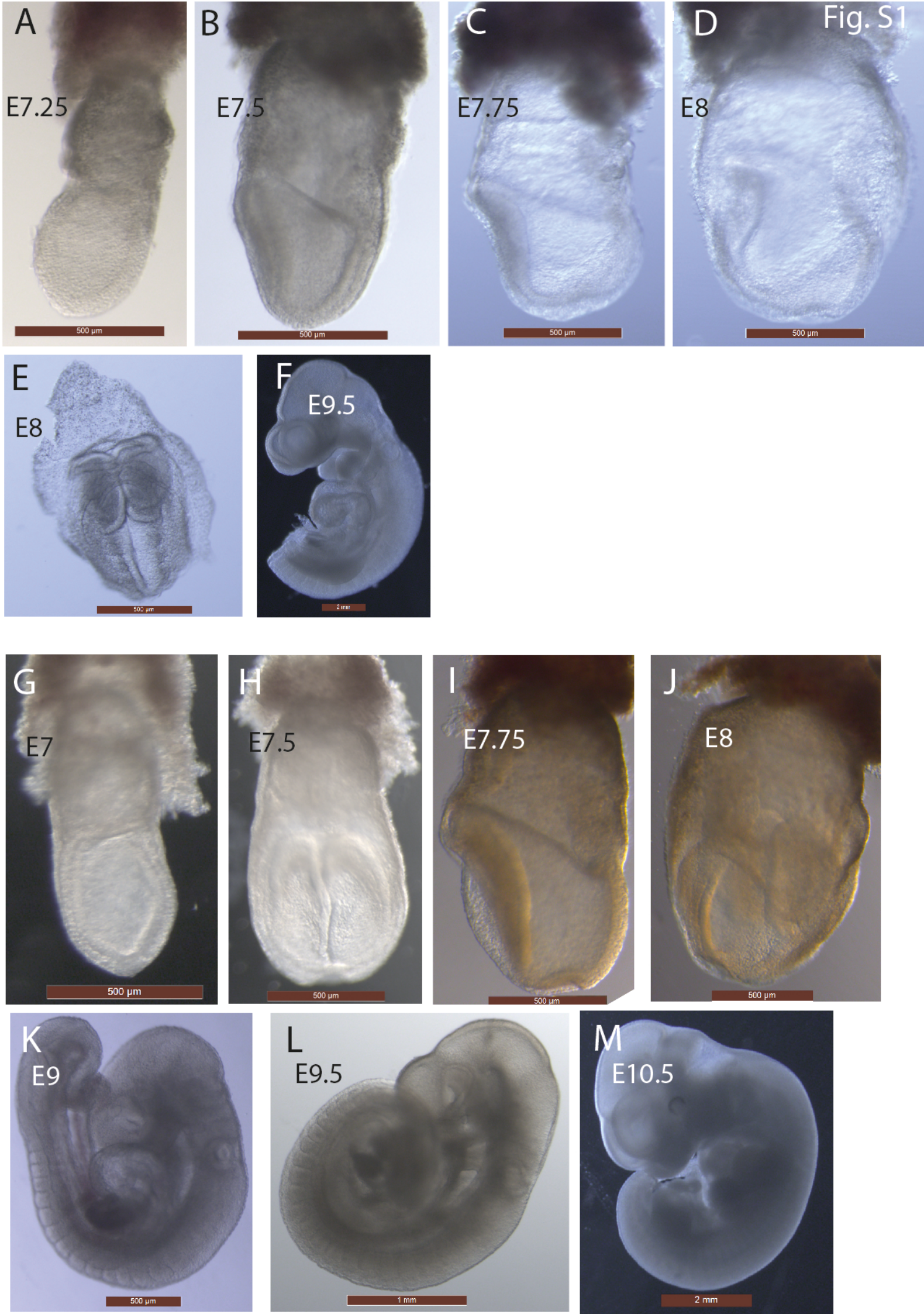

Supplement: Supplementary file 1 — Additional file 1. Brightfield images of the embryos in the movies. A: Additional File 2. B: Additional File 3 and 4; C: Additional File 6 and 7; D: Additional File 8 and 9; E: Additional File 10; F: Additional File 12; G: Additional File 22; H: Additional File 23; I: Additional File 24; J: Additional File 25; K: Additional File 26; L: Additional File 27; M: Additional File 34. [file 12861_2021_239_MOESM1_ESM.pdf]

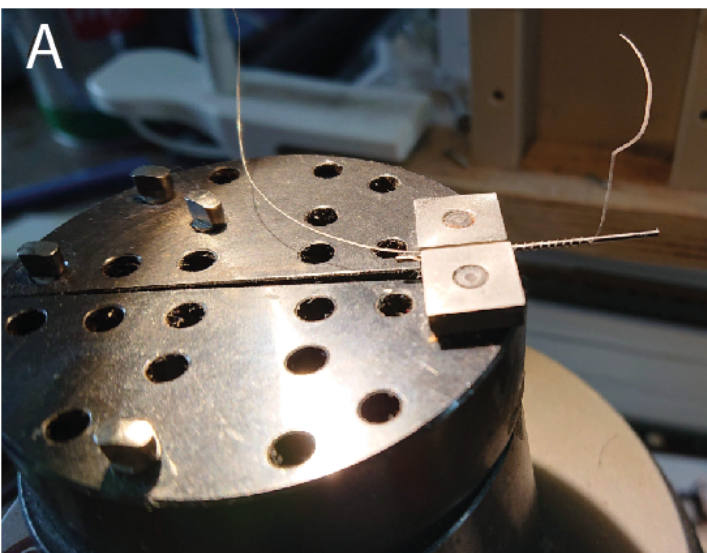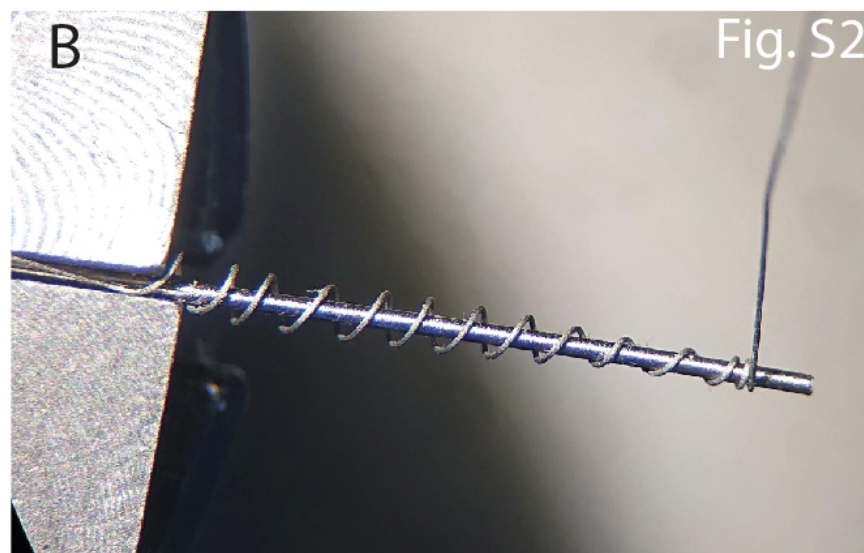

Fig. S2

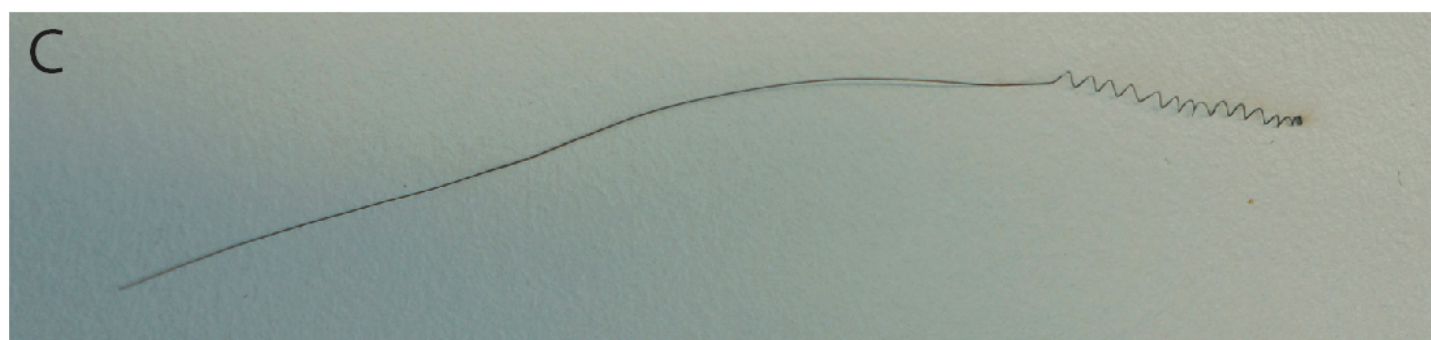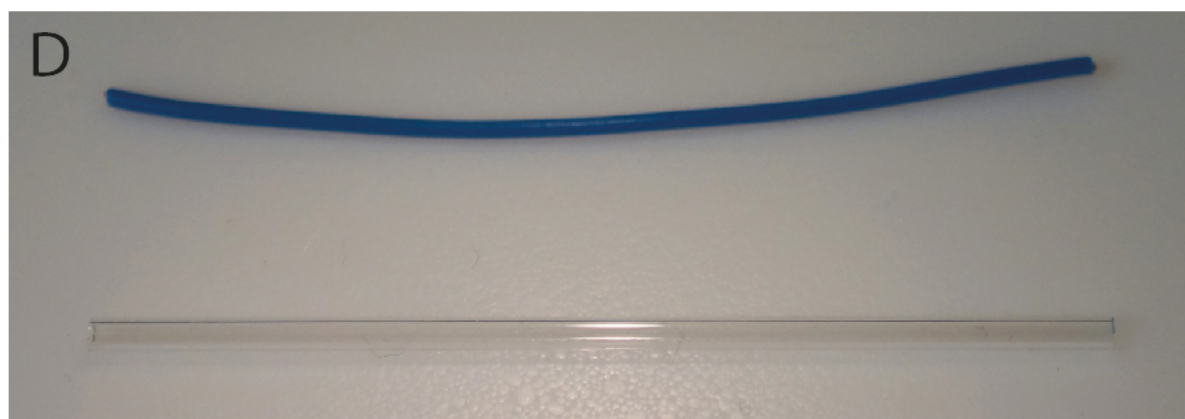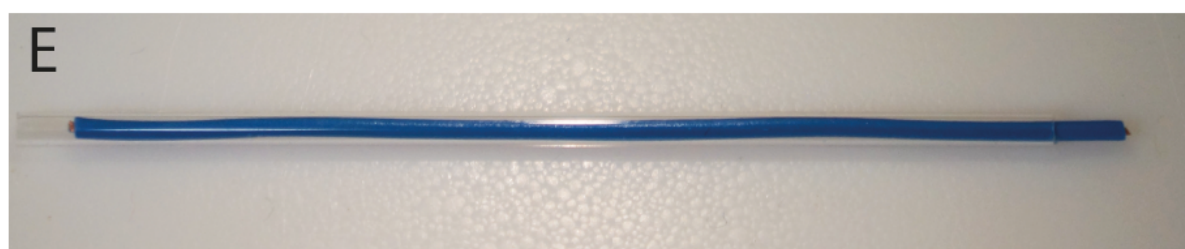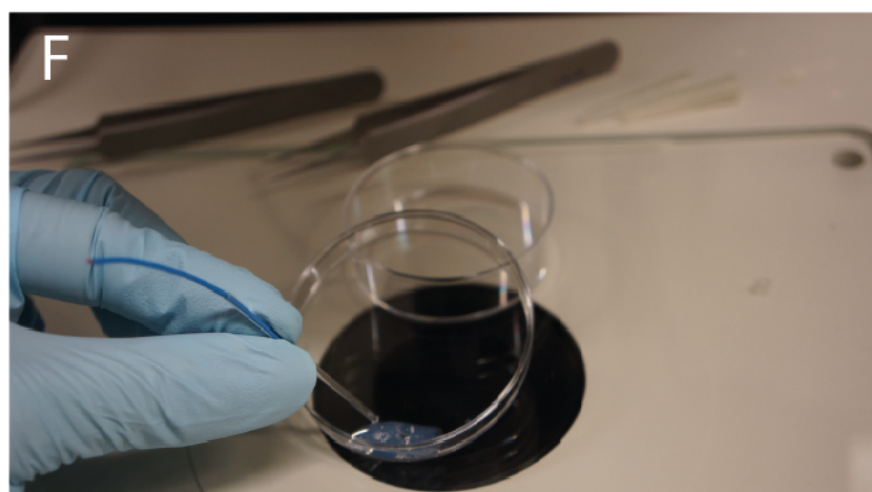

Supplement: Supplementary file 13 — Additional file 13 Mounting an embryo for live imaging with the light sheet microscope The MuVi SPIM light sheet microscope stage holds a glass capillary; a piece of transparent FEP tube (that does not optically interfere with imaging) slides over top of the glass tube and provides a watertight seal: the sample must be held within this. For live imaging of embryos, we developed a new mounting protocol. Embryos were held by their extra-embryonic cones in a single coil of 0.1 mm titanium wire (to minimise toxicity). (A) A large coil was made at one end of a piece of 0.1 mm titanium wire, by winding the wire around the shank of a 0.7 mm drill bit, clamped in a vice. This large coil is designed to anchor the wire into agarose, and to centre the smaller embryo-holding coil. (B, C) To make the embryo-holding coil, the large coil is pulled over the shank of another drill bit, with a diameter suited to the size of the embryo that needs to be mounted (usually between 0.15 and 0.45 mm) and a single coil is made by winding the wire (trimming the excess). (D) Glass capillary with diameter 1.6 mm, and insulated wire (blue) that fits into the glass tube. (E) The blue wire is inserted into the glass capillary. (F) By pulling the end of the blue wire out of the glass capillary, while holding the other end of the glass capillary in 2% molten LMP agarose in CMERL-1066 medium (PANBiotech Cat# P04–84600), agarose is sucked into the glass capillary. [file 12861_2021_239_MOESM13_ESM.pdf]

A

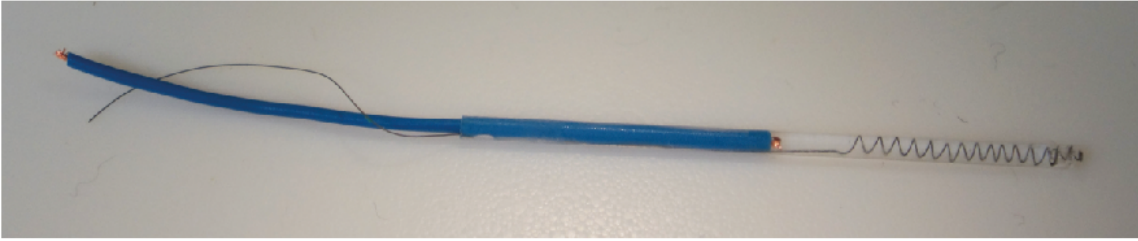

B

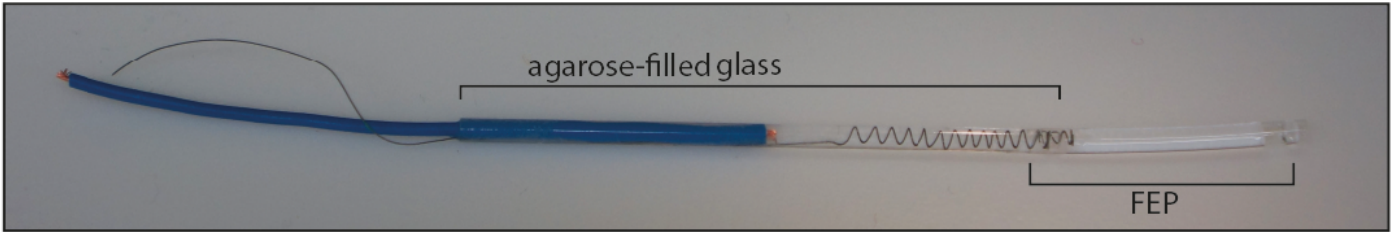

C

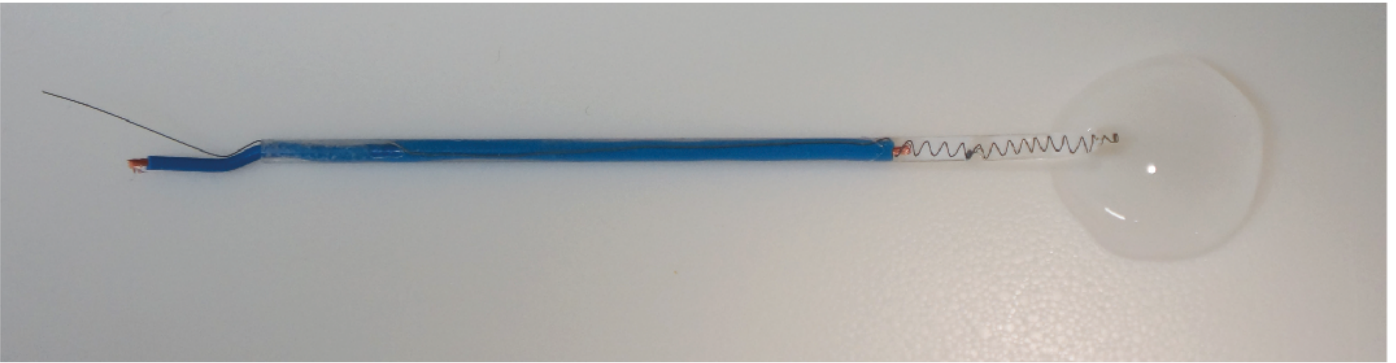

D

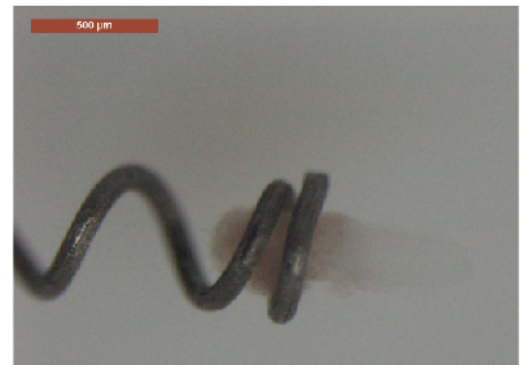

E

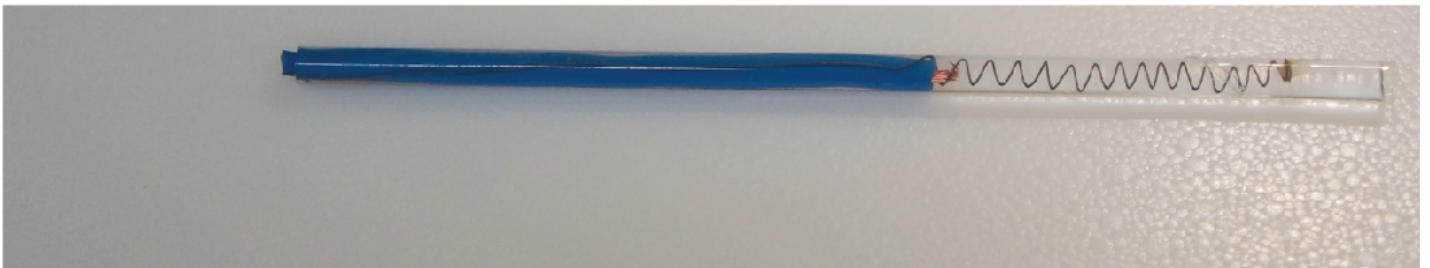

F

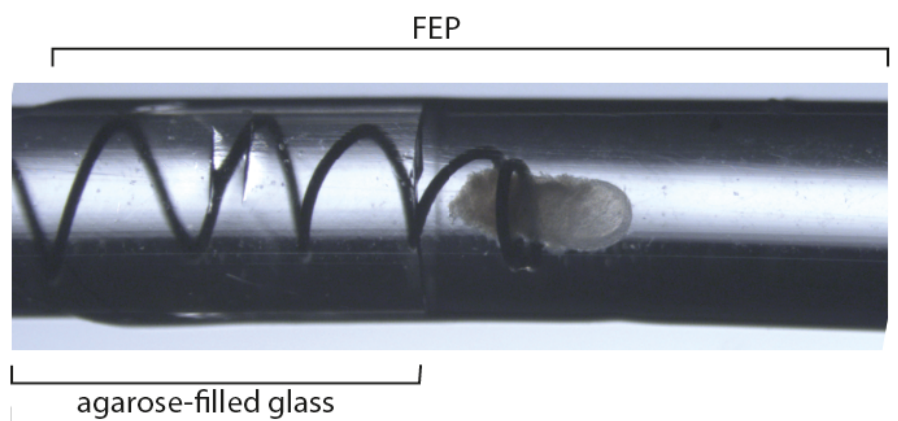

Supplement: Supplementary file 14 — Additional file 14 Mounting an embryo for live imaging with the light sheet microscope (A) The titanium wire is pushed through the glass capillary filled with molten agarose, leaving a small coil sticking out at one end and the bottom of the wire at the other. The blue wire is partially inserted next to the titanium wire. Both wires together will act as a plunger, once the agarose has set, to be able to move the small coil. (B) A piece of FEP tube is filled with rat serum and slid partially over the end of the glass tube. (C) The tip of the FEP tube is immersed in a droplet of rat serum that has been pipetted onto a pre-warmed, water-repellent PTFE plate (to keep the serum as a convenient droplet). The plunger is used to push the tip of the small coil through the FEP tube into the droplet. (D) The embryo is mounted in the top coil inside the droplet, holding it by the detached Reichert’s membrane. (E) The plunger is pulled back slowly, moving the embryo into the FEP capillary filled with rat serum, and the excess titanium and blue wire is trimmed. (F) The embryo is positioned just above the glass tube in the FEP tube. [file 12861_2021_239_MOESM14_ESM.pdf]

Fig. S4

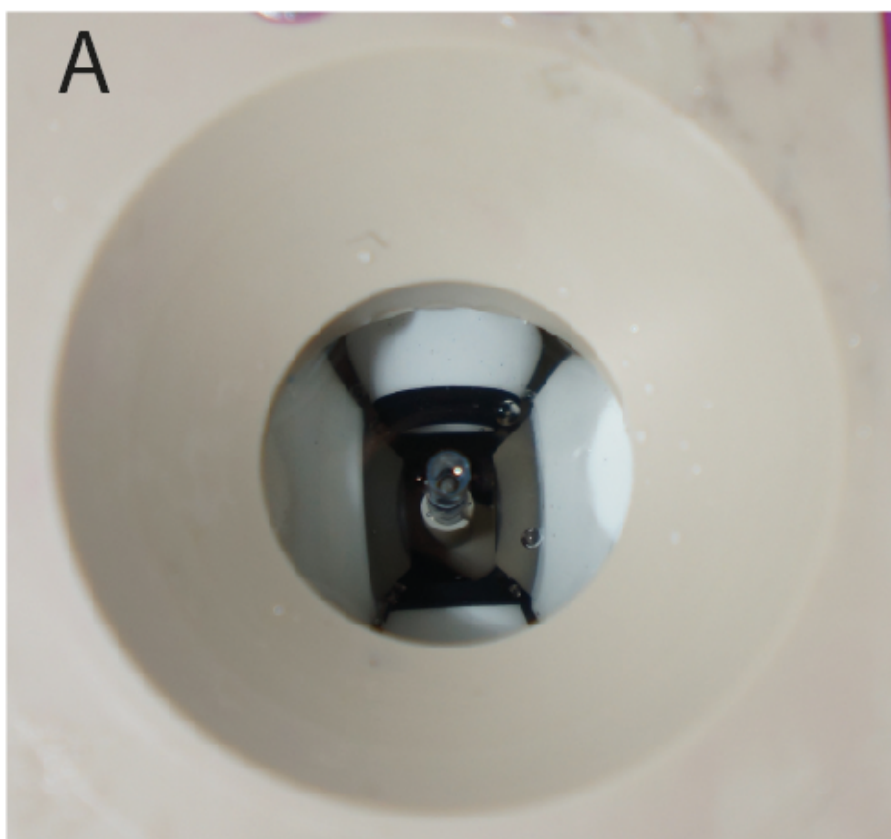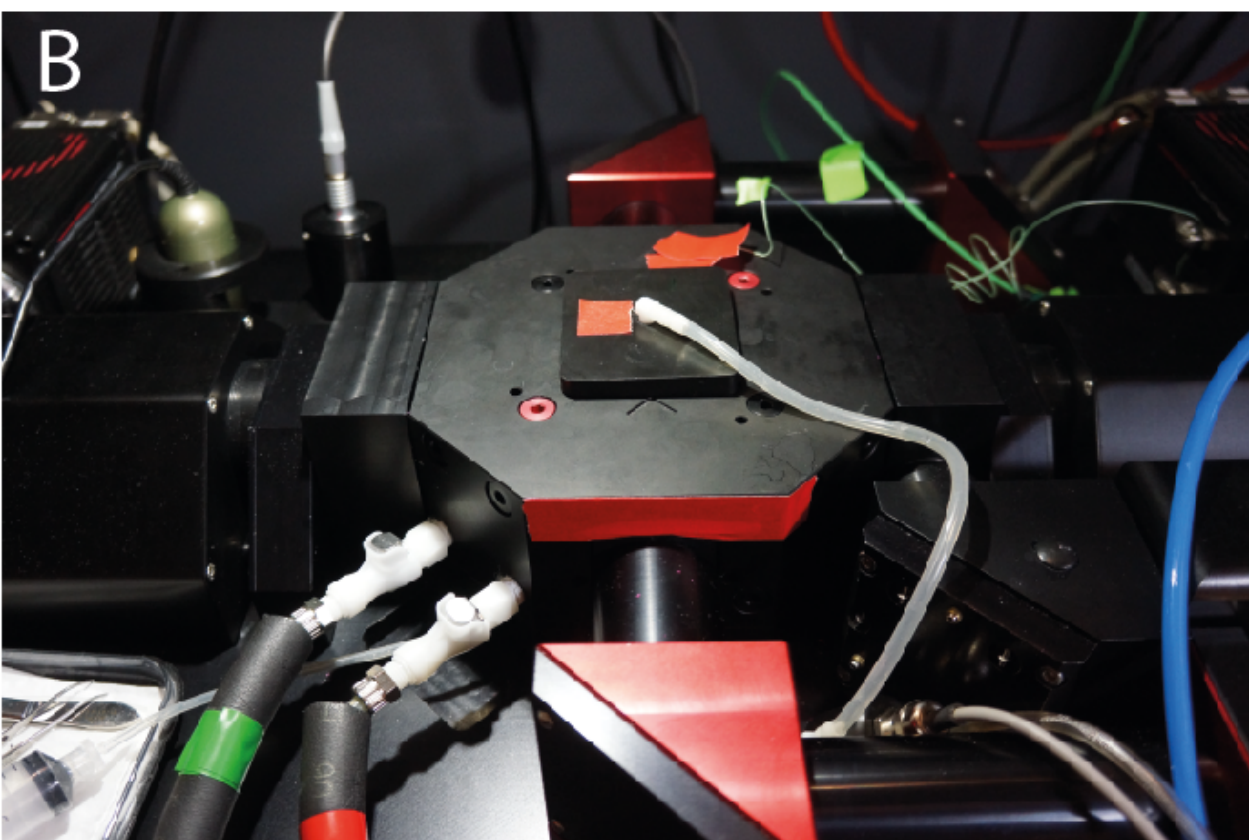

Supplement: Supplementary file 15 — Additional file 15 Mounting an embryo for live imaging with the light sheet microscope (A) The entire contraption is mounted into the pre-warmed, water-filled chamber (with custom-made heater) of the MuVi SPIM microscope, leaving the top of the FEP tube sticking out above the water level. (B) The chamber is closed with a lid that is connected to a gas tank to create the right atmosphere (see Materials and Methods). [file 12861_2021_239_MOESM15_ESM.pdf]
