# Supplementary material for: The migratory pathways of the cells that form the endocardium, dorsal aortae, and head vasculature in the mouse embryo
Source: BMC Dev Biol. 2021 Mar 22;21:8. doi: 10.1186/s12861-021-00239-3 (PMC7986287; doi:10.1186/s12861-021-00239-3)
Supplement: Supplementary file 29 — Additional file 29 Clusters of top and bottom samples (A, B) t-distributed stochastic neighbour embedding (t-SNE) plots, showing clusters of cells of the ‘top’ part (as shown in Fig. 4a) of twenty-five (E7.25-E7.5) embryos, and the localisation of those clusters in the aggregate (B). The Tal1 expressing cells are those of cluster 5, and the Sox17 expressing cells are those of clusters 1 and 6. (C, D) t-SNE plots, showing clusters of cells of the ‘bottom’ part of twenty-five (E7.25-E7.5) embryos, and the localisation of those clusters in the aggregate (D). The Tal1 expressing cells are those of cluster 8 and the Sox17 expressing cells are those of cluster 6. Highly expressed genes in the different clusters can be found in Table S.1 and Table S.2. [file 12861_2021_239_MOESM29_ESM.pdf]

Fig. S5

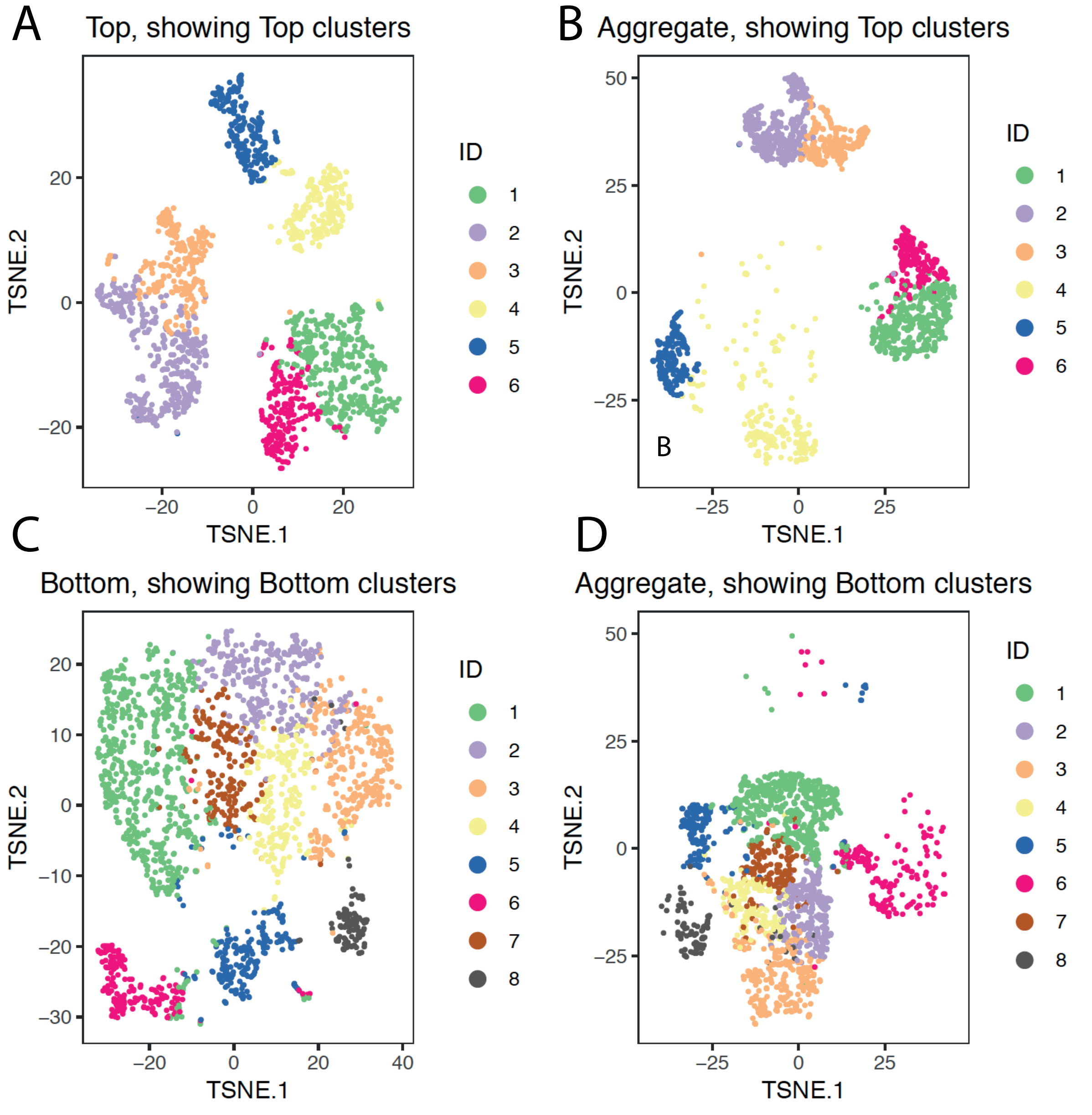
A
Top, showing Top clusters
B
Aggregate, showing Top clusters
C
Bottom, showing Bottom clusters
D
Aggregate, showing Bottom clusters
TSNE.1
TSNE.2
ID
1
2
3
4
5
6
7
8
B
